# Sensor-based precision nutrient and irrigation management enhances the physiological performance, water productivity, and yield of soybean under system of crop intensification

**DOI:** 10.3389/fpls.2023.1282217

**Published:** 2023-12-18

**Authors:** K. S. Sachin, Anchal Dass, Shiva Dhar, G. A. Rajanna, Teekam Singh, Susama Sudhishri, Manjanagouda S. Sannagoudar, Anil K. Choudhary, Hari Lal Kushwaha, B. R. Praveen, Shiv Prasad, Vinod Kumar Sharma, Vijay Pooniya, Prameela Krishnan, Manoj Khanna, Raj Singh, T. Varatharajan, Kavita Kumari, Kadagonda Nithinkumar, Aye-Aye San, Ayekpam Dollina Devi

**Affiliations:** ^1^ ICAR–Indian Agricultural Research Institute, New Delhi, India; ^2^ ICAR-Directorate of Groundnut Research, Regional Station, Ananatpur, Andhra Pradesh, India; ^3^ ICAR-Indian Institute of Seed Science, Regional Station, Bengaluru, India; ^4^ ICAR-Central Potato Research Institute, Shimla, India; ^5^ ICAR-National Dairy Research Institute, Karnal, India; ^6^ ICAR-National Rice Research Institute, Cuttack, India; ^7^ Department of Agricultural Research, Regional Research Centre, Aung Ban, Myanmar

**Keywords:** precision nutrient management, sprinkler irrigation, SPAD, photosynthetic rate, PAR interception, SCI, water productivity, soybean yield

## Abstract

Sensor-based decision tools provide a quick assessment of nutritional and physiological health status of crop, thereby enhancing the crop productivity. Therefore, a 2-year field study was undertaken with precision nutrient and irrigation management under system of crop intensification (SCI) to understand the applicability of sensor-based decision tools in improving the physiological performance, water productivity, and seed yield of soybean crop. The experiment consisted of three irrigation regimes [I_1_: standard flood irrigation at 50% depletion of available soil moisture (DASM) (FI), I_2_: sprinkler irrigation at 80% ET_C_ (crop evapo-transpiration) (Spr 80% ET_C_), and I_3_: sprinkler irrigation at 60% ET_C_ (Spr 60% ET_C_)] assigned in main plots, with five precision nutrient management (PNM) practices{PNM_1_-[SCI protocol], PNM_2_-[RDF, recommended dose of fertilizer: basal dose incorporated (50% N, full dose of P and K)], PNM_3_-[RDF: basal dose point placement (BDP) (50% N, full dose of P and K)], PNM_4_-[75% RDF: BDP (50% N, full dose of P and K)] and PNM_5_-[50% RDF: BDP (50% N, full P and K)]} assigned in sub-plots using a split-plot design with three replications. The remaining 50% N was top-dressed through SPAD assistance for all the PNM practices. Results showed that the adoption of Spr 80% ET_C_ resulted in an increment of 25.6%, 17.6%, 35.4%, and 17.5% in net-photosynthetic rate (P_n_), transpiration rate (T_r_), stomatal conductance (G_s_), and intercellular CO_2_ concentration (C_i_), respectively, over FI. Among PNM plots, adoption of PNM_3_ resulted in a significant (*p*=0.05) improvement in photosynthetic characters like P_n_ (15.69 µ mol CO_2_ m^−2^ s^−1^), T_r_ (7.03 m mol H_2_O m^−2^ s−^1^), G_s_ (0.175 µmol CO_2_ mol^−1^ year^−1^), and C_i_ (271.7 mol H_2_O m^2^ s^−1^). Enhancement in SPAD (27% and 30%) and normalized difference vegetation index (NDVI) (42% and 52%) values were observed with nitrogen (N) top dressing through SPAD-guided nutrient management, helped enhance crop growth indices, coupled with better dry matter partitioning and interception of sunlight. Canopy temperature depression (CTD) in soybean reduced by 3.09–4.66°C due to adoption of sprinkler irrigation. Likewise, Spr 60% ETc recorded highest irrigation water productivity (1.08 kg ha^−1^ m^−3^). However, economic water productivity (27.5 INR ha^−1^ m^−3^) and water-use efficiency (7.6 kg ha^−1^ mm^−1^ day^−1^) of soybean got enhanced under Spr 80% ETc over conventional cultivation. Multiple correlation and PCA showed a positive correlation between physiological, growth, and yield parameters of soybean. Concurrently, the adoption of Spr 80% ET_C_ with PNM_3_ recorded significantly higher grain yield (2.63 t ha^−1^) and biological yield (8.37 t ha^−1^) over other combinations. Thus, the performance of SCI protocols under sprinkler irrigation was found to be superior over conventional practices. Hence, integrating SCI with sensor-based precision nutrient and irrigation management could be a viable option for enhancing the crop productivity and enhance the resource-use efficiency in soybean under similar agro-ecological regions.

## Introduction

Soybean (*Glycine max* (L.) Merill), an introduced crop to India, fits well in all agro-ecological regions of the country (Agarwal et al., 2013; Kumar et al., 2013). In India, it is cultivated in an area of 12.81 million hectares with the annual production of 12.9 million tons (DAFW GOI, 2021). The versatility of soybean to be used in a variety of food sectors, dietary supplements, medicines, and bio-materials will result in a significant increase in demand in the years to come (Rezaei et al., 2002; Raghuvanshi and Bisht, 2010). Yet, India’s average soybean yield is just approximately 1 t ha^−1^, while the global average is 2–3 t ha^−1^. As a result, there is a paucity of soybean grain availability (Bianchi and Szpak, 2017). Input resource conservation has always been the current generation’s top priority if we have to feed the projected 9–10 billion people by 2050 on the same amount of land, water, and other resources (Martens, 2001; Misra, 2014). The advent of new varieties and hybrids with higher harvest index and shorter life cycles has enhanced the agricultural production; concomitantly, they have also increased the ecological footprint by accelerating the depletion of natural resources (Triboi and Triboi-Blondel, 2002; Fess et al., 2011; Singh et al., 2019). With the roots in the system of rice intensification (SRI), the system of crop intensification (SCI) has gained popularity in recent years as a win–win strategy that optimizes crop yields while minimizing ecological footprints and achieving sustainable yields that have fewer inputs by resource requirements (**Table 1**).

**Table 1:** Influence of precise nutrient, irrigation management, and SCI practices on crop performance, available nutrient status, and resource use efficiency.

Sl.no	Management options	Influence	References
**1**	Point placement of N fertilizer	Increased grain yield of wheat 10%–12% compared to surface application	Nayak et al., 2022
**2**	SPAD based N management	Enhances maize yield and saves 30–45 kg N ha^−1^. Improves the chlorophyll content of soybean	Ramesh et al., 2002; Dass et al., 2014; Xiong et al., 2015; Uddling et al., 2017.
**3**	Sensor-based irrigation and depletion of available soil moisture (DASM)-based irrigation schedule	Sensor-based drip irrigation at 125% ETc along with 100% water soluble fertilizer enhanced cane yield. Irrigation at 75% field capacity improved photosynthetic performance and energetics of soybean	Ramesh et al., 2019; Rajanna et al., 2023
**4**	SCI cultivation management	SCI on several field crops have reported 10%–15% higher crop yields, higher net returns, and improved nutrient status of the soil	Dhar et al., 2015; Bhargava et al., 2016; Adhikari et al., 2018; Dass et al., 2023; Singh et al., 2023

Many physiological functions influence crop productivity, such as photosynthesis, transpiration, and stomatal conductance, and have an impact on crop output (Dass and Bhattacharyya, 2017; Pohare et al., 2018). Good agronomic practices can change the physiological performance of the crop by introducing sensible nutrient and irrigation management practices (Singh et al., 2002; Ramesh et al., 2017; Bangre and Singh, 2020; Devaraj, 2020). Furthermore, the application of high rates of fertilizers and irrigation water contributes to greenhouse gas emission, soil salinity, and alkalinity problems, and makes fertile land to be barren that will contribute to climate change (Bennett, 2000; Wang et al., 2002; Ramesh et al., 2017; Jat et al., 2018; Larbi and Green, 2018; Cheng et al., 2021). Therefore, to combat the ill effects of climate change, the use of modern precision tools for nutrient management, such as SPAD meter, GreenSeeker, and leaf color chart (LCC), which correlate the spectral characters of the crop for analyzing the sufficiency or deficiency of particular nutrients in standing crops, could enhance and conserve the available resources (**Table 1**). Precision irrigation, *viz*., automated irrigation, sensor-based and simulation modeling irrigation, is gaining popularity among the farmers in developing countries in view of its ease of operation and water saving under scarce condition. India has more potential for precession irrigation in the future for effective irrigation water management because of the country’s large area under micro-irrigation (13.78 m ha), which includes drip and sprinkler systems (PIB, GOI, 2022). Henceforth, sensor-based tools for irrigation, such as infrared thermometer and soil moisture meter for instant quick profile soil moisture determination, can lead the way for successful in-season precision nutrient and irrigation management with optimum resource efficiency (Bryant et al., 2017; Hou et al., 2018; Wood et al., 2020). However, coming to the adaption of sensor-based irrigation depends on resource base of the farmer, institutional policies, and support from the local government organization through provision of incentives. Proper extension education and spread of knowledge through proper information will help in extending the area under sensor-based irrigation. Furthermore, the precise application of fertilizers through point placement reduces fertilizer requirements and enhances FUE and energetics by maximising crop nutrient uptake (Shafagh et al., 2008; Nayak et al., 2019; Gebre and Earl, 2021). Studies have reported that sprinkler irrigation will enhance the WUE of the crops by 20%–30% and saves water by 30%–45% (Zhao et al., 2009; Li et al., 2019; Solgi et al., 2022). Nevertheless, most of the studies focus on yield and soil fertility status wherein there is not much focus on basic physiological relationships, which finally defines the yield of crop. Hence, the present study throws much insights on growth and physiology of soybean crop with the objectives: to study the applicability of SPAD meter, green seeker, infrared thermometer, and soil moisture meter as an alternative tool to conventional practices for enhancing nutrient and water-use efficiencies and to study the impact of sensor-based tools on net photosynthesis, transpiration, dry-matter accumulations, and other growth indices and their influences on the crop yield.

## Materials and methods

### Site and weather conditions

**Figure 1:**
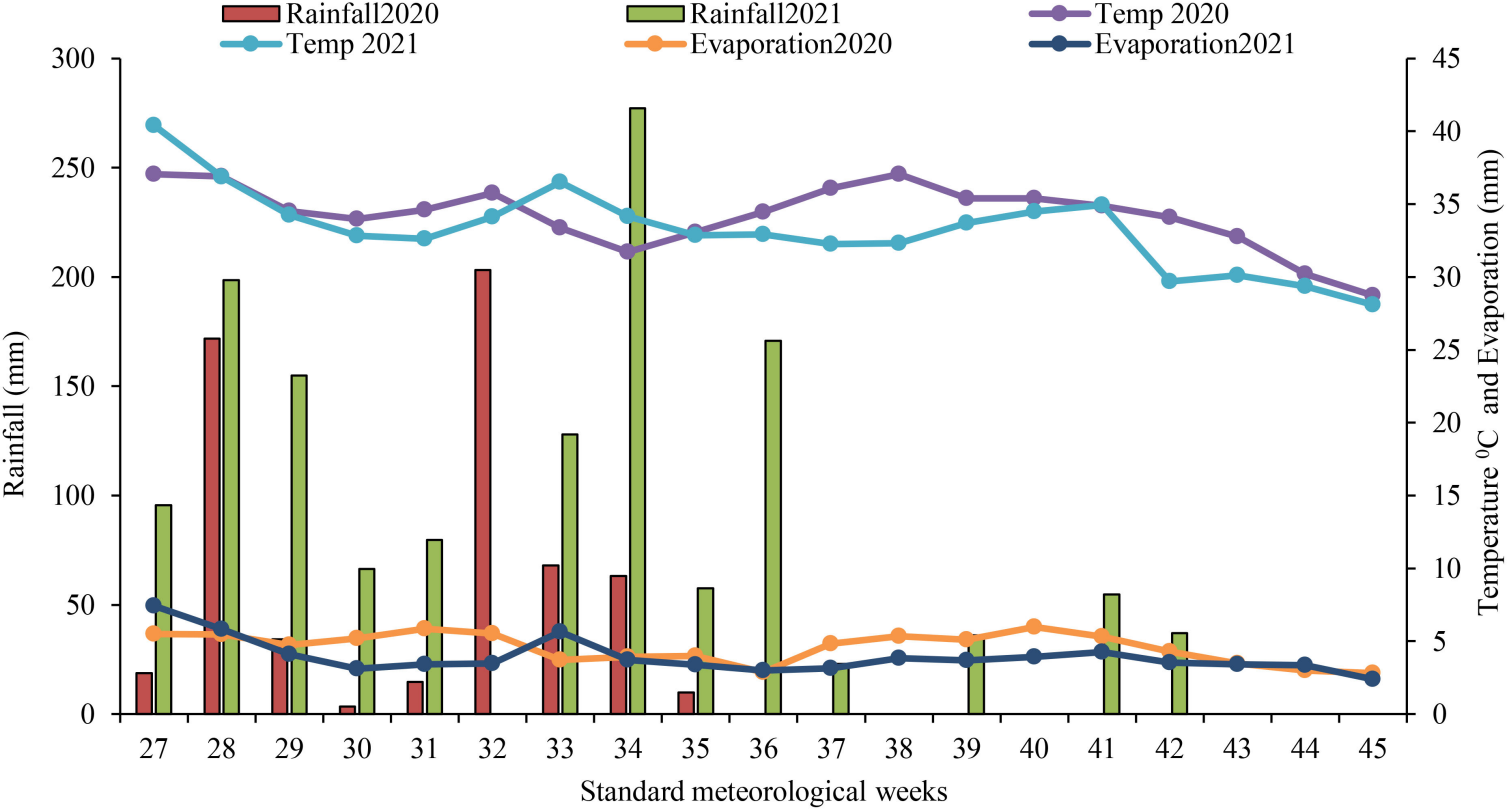
Weekly rainfall, temperature, and evaporation during the kharif 2020 and 2021 (Agro meteorological Observatory, Division of Agricultural Physics, ICAR-IARI, New Delhi).

A 2-year field experiment was conducted in the experimental farm of ICAR-IARI New Delhi, India (latitude of 28°.38′ N and longitude of 77°.09′ E) during the rainy months of the years 2020 and 2021. The experimental site is situated in the semi-arid region of the Indo-Gangetic plains with dry-hot summers and cold winters. The soil of the experimental site was sandy loam in texture and belonged to Typic Ustochrepts. The top 0–15-cm soil layer physicochemical characteristics were estimated using standard analytical techniques (Rana et al., 2014) and presented in **Table 2**. The weekly minimum temperature during the cropping period ranged between 10°C and 28°C, while the maximum temperature ranged from 28°C to 37°C. The cumulative of 587.3 mm was received during kharif 2020 (June to October) and 1,379.4 mm rainfall was received during kharif 2021 (June to October). The cumulative rainfall received during the second season was higher compared to the normal rainfall (**Figure 1**). The daily pan evaporation ranged from 2.3 to 7.4 mm day^−1^. The maximum and minimum relative humidity ranged from 76% to 93% and 32% to 80%, respectively, during the cropping period (**Supplementary Tables S1, S2**).

**Table 2:** Physicochemical properties of the soil at the experimental field.

Particulars	Values
Mechanical analysis	
Sand (%)	64.28
Silt (%)	20.56
Clay (%)	13.98
Textural class	Sandy loam
Physical Properties	
Bulk density (Mg m^−3^)	1.40
Chemical properties	
Soil pH (1:2.5 soil:water ratio)	7.8
EC (1:2.5 soil:water ratio) (dS m^−1^)	0.32
Soil organic carbon (%)	0.53
Available N (kg ha^−1^) (KMnO_4_–oxidizable N)	195.3
Available P (kg ha^−1^) (0.5M NaHCO_3_ extractable P)	12.0
Available K (kg ha^−1^) (0.1N NH_4_OAc exchangeable K)	262.8

### Study details

The present study was carried out in a split-plot design, comprising three main plots (315 m^2^ each) and five sub-plots (21 m^2^ each). The main plots were assigned to irrigation (I): I_1_, 50% depletion of available soil moisture (DASM) (FI); I_2_, sprinkler irrigation at 80% ET_C_ (crop evapotranspiration) (Spr 80% ET_c_), and iii), sprinkler irrigation at 60% ET_C_ (Spr 60% ETc). The total gross plot area with three replications were 945 m^2^.The sub-plots were allotted with precision nutrient management (PNM_1–5_) as given in **Table 3**. Portable sprinklers (Spr 80% ET_c_ and Spr 60% ETc) were used for irrigating the crops. In addition, there was an absolute control plot (conventional management practices both for irrigation and nutrient management). The soybean–wheat cropping system has been followed in the present study under the system of crop intensification (SCI) practices.

**Table 3:** Details of treatment combination of precision nutrient and irrigation management adopted in soybean under system of crop intensification (SCI).

Precision nutrient management (PNM)	Short forms	Irrigation management (I)		
		Standard irrigation (Surface method at 50% of DASM)	Sprinkler irrigation at 80% of ET_C_	Sprinkler irrigation at 60% of ET_C_
		I_1_	I_2_	I_3_
SCI protocol for nutrient management	PNM_1_	+	+	+
RDF: basal dose incorporated (50% of N Full of P + K) + 50% of N SPAD based	PNM_2_	+	+	+
RDF: basal dose point placement (50% of N Full of P + K) + (50% of N SPAD based)	PNM_3_	+	+	+
75% RDF: basal dose point placement (50% of N Full of P + K) + (50% of N SPAD based)	PNM_4_	+	+	+
50% RDF: basal-point placement (50% of N Full of P + K) + (50% of N SPAD based)	PNM_5_	+	+	+
**Control**	C	−	−	−

RDF, recommended dose of fertilizer; SPAD, soil plant analysis development; DASM, depletion of available soil moisture; ET_C_, crop evapotranspiration; N, nitrogen; P, phosphorus; K, potassium.

“+” symbol represents combination two factors irrigation (I) and precision nutrient managment (PNM).

“−” Symbol represents in control neither sprinkler irrigation nor precision nutrient management practices were adopted.

The standard package of practices for system of crop intensification (SCI) cultivation of soybean was adopted as earlier used by Singh et al. (2023). Under SCI, seeds were treated with the solution made from the combination of vermicompost (2.5 kg), 5 L of cow urine, and 2.5 kg of jaggery in 20–25 L of hot water (60°C). The vermicompost and water solution were prepared separately, and after cooling, both solutions were mixed, and the soybean seeds were soaked in the mixed solution for 2 h. Furthermore, the seeds were separated from the solution by filtering. Then, the seeds were treated with *Rhizobium* and phosphorus-solubilizing bacteria (PSB) and allowed them to shade dry overnight; then, the pre-germinated seeds were used for sowing. Two pre-germinated seeds were dibbled at 30×30 cm per hill by hand sowing. The nutrients were applied as per the treatment given in **Table 4**. After 7 days of sowing, gap filling (pre-germinated seeds) was done to maintain the ideal plant population. The seed treatment was uniformly followed in all the treatments. In SCI nutrient management (PNM_1_), the recommended amount of nutrients was administered during field preparation using vermicompost at 2.5 t ha^−1^ treated with *Trichoderma* (2.5 kg t^−1^), and the remaining amount of phosphorus was supplied through single super phosphate at 250 kg ha^−1^. The recommended dose of fertilizer (RDF) for soybean under New Delhi region based on soil-test crop response (STCR)-based recommendation is 52:72:40 (N:P_2_O_5_:K_2_O kg ha^−1^), respectively. For PNM_2–5_, STCR-based RDF was practiced. The source of N and P_2_O_5_ fertilizer was di-ammonium phosphate (DAP), and K_2_O fertilizer was muriate of potash (MOP). Based on treatment combinations, N dose was split into two parts, one part was applied basally at the time of sowing along with full amount of P and K, for PNM_2–5_ treatments. The remaining 50% of N was top dressed based on the SPAD readings; SPAD value ≤ 30 was taken as standard reference reading for top dressing of N in soybean (Dass et al., 2022; Rajanna et al., 2022a), The fertilizers were placed into soil in two ways: (i) broadcasting and ii) point placement (fertilizers placed manually around root zone of plant in circular manner). Under the control treatment, the crop was grown with standard package of practices. The total amount of nutrients supplied for each PNM is presented in **Table 3**. Hand hoeing and manual weeding were done twice at 20 DAS and 40 DAS; standard management practices were followed for other pest and disease management.

**Table 4:** Treatment wise fertilizer application (kg ha^−1^) in soybean.

PNM levels	Total amount of nutrients added (kg ha^−1^)		
	N	P_2_O_5_	K_2_O
PNM_1_	37	53	34
PNM_2_	52	72	40
PNM_3_	52	72	40
PNM_4_	39	72	40
PNM_5_	26	72	40
C	25	75	25

For details description on PNM_1–5_, refer to **Table 2**; N, nitrogen; P_2_O_5_, phosphorus; K_2_O, potassium.

#### Water use and soil moisture measurement

**Figure 2:**
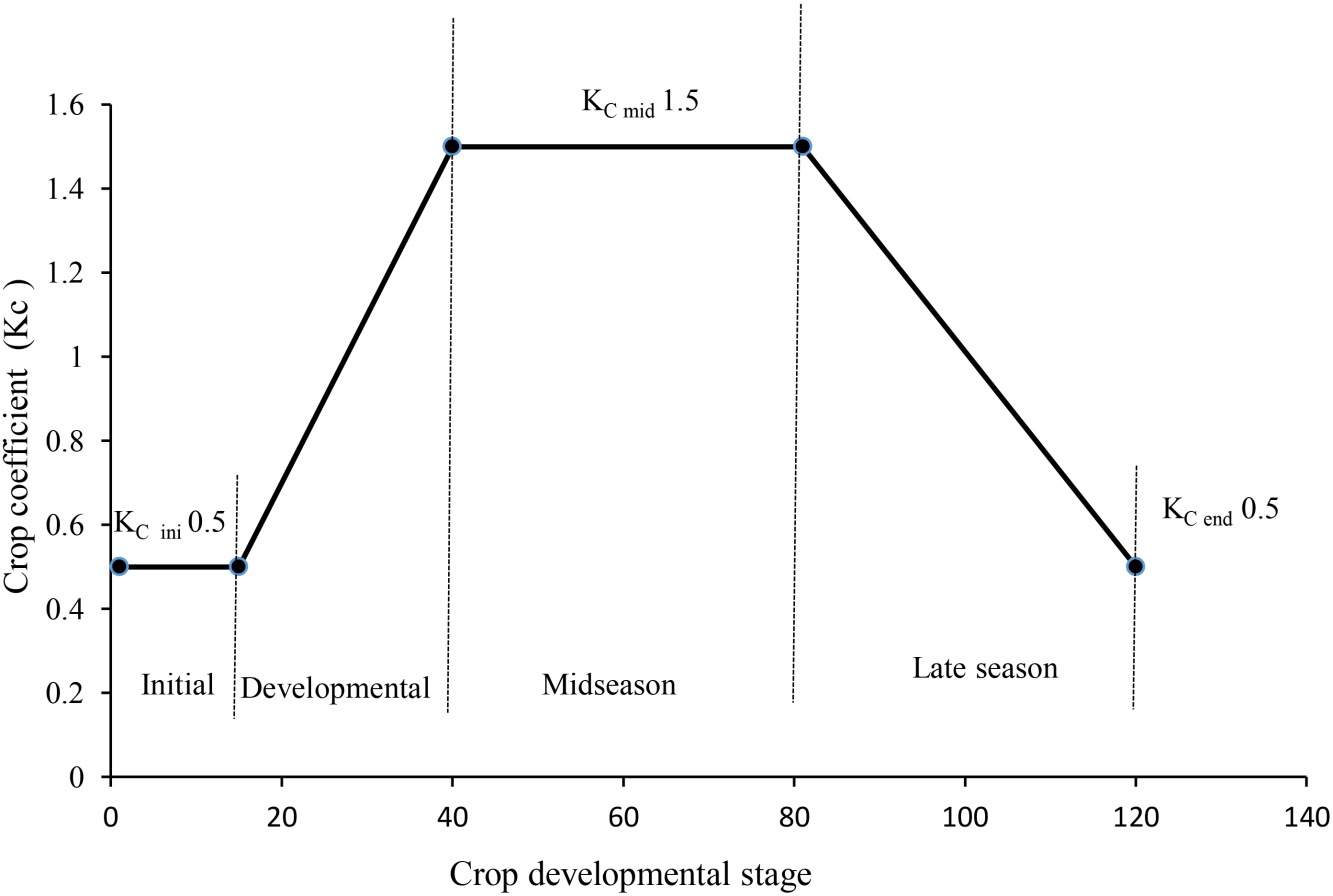
Soybean crop coefficient (KC) values for determining the ETc for scheduling irrigation.


A soil moisture meter (Delta-T Devices Ltd., Burwell, Cambridge, CB5 0EJ, UK) was used to measure the volumetric soil water content (v/v) to monitor depletion of available soil moisture. Each plot’s volumetric changes in soil moisture were tracked using a soil moisture sensor at intervals of 0–10 cm, 10–20 cm, 20–30 cm, and 30–40 cm soil depth. Soil moisture sensor (access) tubes were inserted into soil up to 1 m depth between the crop rows using a post-hole auger, containing one tube in each main plot. The Delta-T (DL-6) PR 2 soil moisture probe (Delta-T Devices Ltd., Burwell, Cambridge, CB5 0EJ, UK) was used to measure the volumetric moisture content of the soil at various crop growth stages. In FI, when soil moisture approaches 50% depletion of available soil moisture, irrigation was commissioned. In each irrigation, 5 cm depth equivalent amount of water was applied. For scheduling Spr 80% ETc and Spr 60% ETC, sprinkler irrigation ET_C_ was computed by using ET_0_ (reference evapotranspiration) and K_c_ (Crop coefficient). ET_0_ was worked out by using the Penman -Monteith method to determine the K_C_. The stages of crop development are identified as early stage (Kc ini), development stage, mid-season stage (Kc mid), and late season stage (Kc end), along with their lengths and appropriate Kc coefficients. The chosen Kc coefficients are then adjusted for the frequency of wetting or climatic conditions for each stage of the soybean. Finally, the Kc curve was constructed against different stages of crop growth for any time during the growing season (**Figure 2**). ET_c_ was calculated as the product of ET_0_ and Kc (Allen et al., 1998).

(1) $$\text{ETc}={\text{ET}}_{\circ }\times \text{Kc}$$

A total of four irrigations were given to the crops in both the cropping cycles. Sprinkler irrigation was scheduled twice a week (especially during dry spells) as determined by the ETc of the crop accordingly 80% ETc and 60% ETc.

#### SPAD reading (chlorophyll content) and normalized difference vegetation index

Five healthy tagged plants were used to measure the chlorophyll content or leaves greenness by placing the SPAD meter (Konica-Minolta SPAD-502, Osaka, Japan) on the middle part of the fully expanded trifoliate leaf, avoiding the main vein. Five readings from different leaves within the plant were taken, and the average SPAD reading was calculated for each plant. The SPAD readings were taken regularly at weekly intervals between 30–45 DAS and 60–90 DAS and used for analysis.

The NDVI was calculated using a handheld optical crop sensor Trimble GreenSeeker^®^ (Trimble, Sunnyvale, CA, USA). To achieve the average NDVI values, the GreenSeeker^®^ device was moved along the rows of crops while being held at the same height above the crop canopy (50 cm).The GreenSeeker^®^ emits near-infrared (NIR) and red light toward the vegetation canopy. Different wavelengths of light were absorbed based on the chlorophyll content in the plant leaves and other plant pigments. The amount and spectral properties of the reflected light depend on the health and physiological status of the plants, including factors such as leaf chlorophyll content, and canopy structure. The NDVI readings taken regularly at weekly intervals from 30 to 45 DAS and 60 to 90 DAS were used for analysis.

#### Photosynthetic characters and net assimilation rate

The fully opened index leaves of the soybean plant were put in the sensor chamber of the instrument i.e., infrared gas analyzer (IRGA) [LI 6400 XT], a portable photosynthetic system. The photosynthetic characteristics of the crop were measured during the flowering stage (R2) of the crop on a bright sunny day between 900 hours and 1100 hours. The CO_2_ concentration was maintained at ambient, the air flowrate through the chamber was 500 mol s^−1^, and the relative humidity was between 70% and 75%. Photosynthetic characters recorded include the following: i) net photosynthetic rate (Pn)—amount of CO_2_ consumed by leaves per unit area per unit time (µ mol CO_2_ m^−2^ s−^1^); ii) transpiration rate (Tr)—the amount of water consumed by leaves per unit area per unit time (m mol H_2_O m^−2^ s^−1^); iii) stomatal conductance (Gs)—gas exchange of stomata per unit area per unit time (mol H_2_O m^−2^ s^−1^); and iv) inter-cellular CO_2_ concentration (i_C_)—concentration of CO_2_ inside leaf (mol H_2_O m^2^ s^−1^).

#### Canopy temperature depression

The relative temperature of the CTD to the surrounding air was determined using a handheld infrared thermometer; five measurements of canopy temperature were taken from each experimental plot between12:00 and 14:00 on sunny days only. The temperature of the surrounding air was also measured simultaneously. The following formula was used to compute the canopy temperature depression.


(2) CTD (°C)=air temperature(°C)−canopy temperature(°C)

#### Photosynthetically active radiation

Photosynthetically active radiation (PAR) denotes the fraction of solar radiation, i.e., the number of moles of photons in the radiant energy in a specific spectral range (400–700 nm). It was measured using the canopy analyzer LP-80 Accu PAR Line Quantum Sensor of 1 m length connected with a data logger and expressed in μmol (photons) m^−2^ s^−1^. PAR was measured by placing the Line Quantum Sensor in between the plant rows to record transmitted solar radiation (at bottom of the crop canopy), while incident solar radiation was measured at top of the crop canopy. Five readings per plot were recorded at 30–45 DAS and 60–90 DAS between 12:00 and 13:00 on a clear sunny day.


PAR interception=incident solar radiation−transmitted solar radiation

#### Crop growth indices

Dry matter accumulation was calculated by taking dry matter at 60 DAS from the net plot area and finally expressed in g m^−2^. Five representative plants from plot were randomly sampled at 30 and 60 DAS and oven-dried at 60–70°C. Based on plant dry weight, crop growth indices, such as leaf area index, RGR, CGR, and NAR, were worked out using Equations 3–5 as given by Watson (1952);


(3) $$\text{CGR }\left({\text{g m}}^{-2}{\text{day}}^{-1}\right)=\;\left(\frac{{\text{W}}_{2}-{\text{W}}_{1}}{{\text{T}}_{2}-{\text{T}}_{1}}\right)\left(\frac{1}{\text{S}}\right)$$

(4) $$\text{RGR }\left({\text{mg g}}^{-1}{\text{day}}^{-1}\right)=\;\frac{\left({\text{LnW}}_{2}-{\text{LnW}}_{1}\right)}{\left({\text{T}}_{2}-{\text{T}}_{1}\right)}$$

(5) $$\text{NAR }\left({\text{mg cm}}^{-2}{\text{day}}^{-1}\right)=\;\left(\frac{{\text{W}}_{2}-{\text{W}}_{1}}{{\text{LA}}_{2}-{\text{LA}}_{1}}\right)\left(\frac{{\text{LnLA}}_{2}-{\text{LnLA}}_{1}}{{\text{T}}_{2}-{\text{T}}_{1}}\right)$$

where W_1_ and W_2_ are dry weights of plant, LA_1_ and LA_2_ are the leaf area, T_1_ and T_2_ are the time intervals in days, and S is the land area occupied by the plants.

The leaf area was measured using an LI-3100C leaf area meter (Li-COR, Lincoln, NE, USA) as cm^2^ plant^−1^, and the leaf area index (LAI) was calculated as per Rana et al. (2014).

#### Yield estimation

The crop was harvested from the net-plot area (18.3 m^2^) leaving behind the two border rows and dried in the field for 7–10 days, and the whole biomass weight was recorded for each treatment and designated as biological yield. The dried samples were threshed plotwise by using Pullman thresher.

#### Water productivity

For the purpose of estimating water productivity, the irrigation water provided to each plot was measured, and different calculations were made using the rainfall data gathered from the ICAR-Indian Agricultural Research Institute’s meteorological observatory in New Delhi.

(a) Irrigation water productivity (IWP) (kg ha^−1^ m^−3^).

The computation of irrigation water productivity for soybean crop was done utilizing the formula used by Dass et al. (2013).


(6) $$\text{Irrigation water productivity }\left(\text{IWP}\right)\;=\frac{\text{Grain yield }\left({\text{kg ha}}^{-1}\right)}{\text{Irrigation water use }\left({\text{m}}^{3}\right)}$$

(b) Economic water productivity (INR ha^−1^ m^−3^).

The calculation of economic water productivity (EWP) for soybean was carried out following the methodology described by Igbadun et al. (2006).


(7) $$\text{Economic water productivity }\left(\text{EWP}\right)=\frac{\text{ Market price of grains }\left({\text{INR kg}}^{-1}\right)\;\times \text{ Grain yield }\left({\text{kg ha}}^{-1}\right)}{\text{Total amount of seasonal water used in ha}–\text{mm}}$$

*(c) Water-use efficiency (WUE) (kg ha^–1^ mm^–1^ day^−1^)*.

Water-use efficiency was worked out as the ratio of yield to evapotranspiration of crop (Dass et al., 2013).


(8) $$\text{WUE}=\frac{\text{ Yield }\left({\text{kg ha}}^{-1}\right)}{\text{Evapotranspiration }\left({\text{mm day}}^{-1}\right)}$$

#### Statistical analysis

The difference between the treatments was statistically analyzed through ANOVA by using JMP^®^ software from SAS. The significant difference between the two treatment mean values was determined by using least significance difference (LSD) by performing Tukey’s honestly significant difference (HSD) test (*p*<0.005) and indicated by different letters. A comparison was made between control versus SCI and control versus the rest of the treatment combinations to compare the performance of crop under SCI versus conventional cultivation. For better understanding of the relationship between different growth, physiological, and yield parameters, multiple variate analysis (MVA) and principle component analysis (PCA) were performed using JMP^®^software.

## Results

### Irrigation effects

The adoption of ETc-based sprinkler irrigation significantly enhanced photosynthetic characters of soybean during 2020 and 2021. Net photosynthetic rate (P_n_) at flowering stage under sprinkler irrigation (Spr 80% Etc) showed 25.6% and 42.5% higher Pn value compared to FI (standard flood irrigation at 50% DASM) during 2020 and 2021, respectively (**Table 5**). Likewise, the adoption of Spr 80% Etc irrigation schedule enhanced transpiration rate (Tr) from 5.59 to 6.42 m mol H_2_O m^2^ s^−1^ during both study years. Similarly, the practice of Spr 80% ETc exhibited significantly (*p*>0.05) higher Tr (6.66 and 7.33 m mol H_2_O m^2^ s^−1^), stomatal conductance (Gs) (0.17 and 0.21 mol CO_2_ mol^−1^ year^−1^) and intercellular CO_2_ concentration (Ci) (246.6 and 319.4 mol H_2_O m^2^ s^−1^) than flood irrigation during both crop seasons, respectively. However, FI and Spr 60% ETc did not show any significant difference between them for Gs and Ci in both crop seasons. In both years, there was a notable increase of 14.9% and 16.3% in NAR in soybean under Spr 80% ETc compared to FI.

**Table 5:** Effect of precision nutrient and irrigation management on photosynthetic character, *viz.*, net photosynthetic rate Pn, transpiration rate Tr, stomatal conductance Gs, inter-cellular CO_2_ concentration Ci, and net assimilation rate at flowering stage of soybean under SCI.

Treatments		Net photosynthetic rate (P_n_) (µmole CO_2_ m^2^ s^−1^)			Transpiration rate (T_r_) (m mol H_2_O m^2^ s^−1^)			Stomatal conductance (G_s_) (µmol CO_2_ mol^−1^ year^−1^)					Inter-cellular CO_2_ concentration (C_i_) (mol H_2_O m^2^ s^−1^)				Net assimilation rate (NAR) (mg cm^−2^ day^−1^)	
		2020	2021		2020	2021		2020			2021		2020		2020		2020	2021
*Irrigation management*																		
I_1_		12.5^b^	10.45^b^		5.59^b^	6.27^b^		0.13^b^			0.15^b^		210.2^b^		271.9^b^		9.20^b^	8.10^b^
I_2_		15.68^a^	15.08^a^		6.66^a^	7.33^a^		0.17^a^			0.21^a^		246.8^a^		319.4^a^		10.82^a^	9.68^a^
I_3_		14.19^ab^	13.38^a^		6.43^a^	6.42^ab^		0.14^ab^			0.17^b^		221.0^b^		294.1^b^		10.01^ab^	8.92^ab^
SEm ±		0.37	0.35		0.12	0.13		0.01			0.01		4.53		4.89		0.24	0.20
LSD (P=0.05)		1.46	1.38		0.33	0.74		0.02			0.03		17.79		19.20		0.94	0.77
*Nutrient management*																		
PNM_1_		14.17^b^	13.38^b^		6.28^b^	6.99^ab^		0.15^a^			0.18^ab^		231.7^ab^		299.3^a^		10.33^ab^	9.29^ab^
PNM_2_		14.37^b^	13.12^b^		6.14^bc^	6.66^ab^		0.14^ab^			0.17^ab^		223.6^b^		298.3^a^		9.99^ab^	9.36^b^
PNM_3_		15.90^a^	15.48^a^		6.89^a^	7.17^a^		0.16^a^			0.19^a^		238.9^a^		304.4^a^		10.71^a^	9.69^a^
PNM_4_		13.75^bc^	12.50^b^		6.06^bc^	6.37^ab^		0.14^ab^			0.18^ab^		227.3^b^		291.5^ab^		9.89^b^	8.74^b^
PNM_5_		12.42^c^	10.36^c^		5.75^C^	6.17^b^		0.13^b^			0.17^b^		208.5^c^		282.1^b^		9.12^c^	8.34^c^
SEm ±		0.33	0.32		0.10	0.20		0.01			0.01		2.73		4.28		0.22	0.19
LSD (***p*** =0.05)		0.97	0.92		0.29	0.58		0.02			0.02		7.98		12.52		0.65	0.57
I×PNM		S	S		NS	NS		NS			NS		NS		NS		S	S
Control vs. SCI																		
Control		13.00^b^	9.45^b^		6.07^b^	6.10^b^		0.12^b^			0.14^b^		225.7^a^		283.7^b^		8.93^b^	9.27^a^
SCI		14.12^a^	12.97^a^		6.22^a^	6.67^a^		0.14^a^			0.18^a^		226.0^a^		295.1^a^		10.01^a^	9.08^a^
SEm ±		0.15	0.14		0.04	0.09		0.002			0.002		1.22		1.91		0.10	0.09
LSD (***p*** =0.05)		0.43	0.41		0.13	0.26		0.007			0.006		3.57		5.60		0.29	NS

I_1_, standard flood irrigation 50%DASM (FI); I_2_, sprinkler irrigation at 80% ET_C_ (Spr 80% ETc); I_3_, sprinkler irrigation at 60% ET_C_ (Spr 60% ETc). For a detailed description of PNM_1–5_, refer to **Table 2**. Values with different superscript letters in a column are significantly (p<0.05) different as determined by Tukey’s honest significant difference test. NS-non significant, whereas S indicates Significant at (p<0.05).

**Figure 3:**
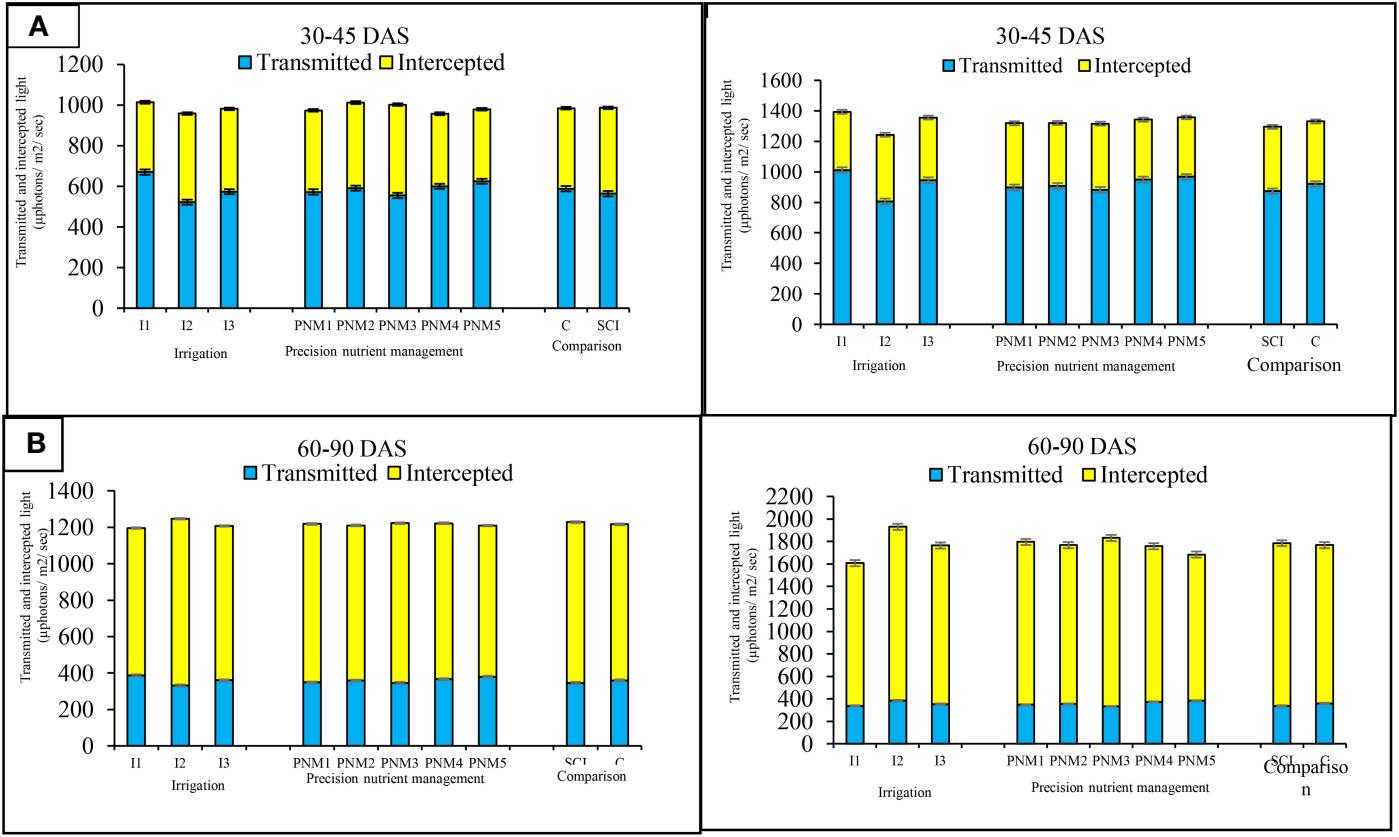
Effect of precision nutrient and irrigation management on transmission and interception photosynthetically active radiation (PAR) by soybean crop canopy at 30–45 DAS **(A)** and 60–90 DAS **(B)** during 2020 and 2021. For a detailed description of I (irrigation) and PNM (precision nutrient management), refer to **Table 2**. Any treatment difference that exceeds the range of the bar within a given year is significantly different, as indicated by the LSD 0.05 bar above each column.

The SPAD values varied from 27.6 to 31.7 at 30–45 DAS among irrigation regimes across study years. The treatment Spr 80% ETc recorded significantly (*p*=0.05) higher SPAD values over FI (**Table 6**). In general, the chlorophyll content (SPAD) followed the order of Spr 80% ETc > Spr 60% ETc >FI. Using sprinkler irrigation at Spr 80% ETc increased NDVI values by 23.6%–27.7% and 12.7%–16.1% at 30–45 DAS and 60–90 DAS, respectively, over FI treatment. There was a reduction in canopy temperature of soybean ranging from 3.12°C to 4.44°C over the entire crop growth duration (**Table 6**). The CTD with Spr 80% ETc was significantly (*p*=0.05) lower 24.4%–25.9% and 33.9%–42.3% at 30–45 DAS and 60–90 DAS, respectively over standard flood irrigation plots. Similarly, photo synthetically active radiation (PAR) and transmitted and intercepted light were recorded at 30–45 DAS and 60–90 DAS period (**Figures 3A, B**). At 30–45 DAS, FI exhibited significantly greater (*p*=0.05) light transmittance vis-à-vis less light interception by the crop canopy than others. With the advancement of the crop age (60–90 DAS), greater crop canopy resulted more light interception vis-à-vis less transmission of the incident light through the canopy. PAR interception in Spr 80% ETc showed an increment of 14.4%–27.0% and 13.2%–21.8% at 30–45 DAS and 60–90 DAS over FI.

**Table 6:** Effect of precision nutrient and irrigation management on SPAD, normalized difference vegetation index (NDVI), and canopy temperature depression (CTD) at 30–45 DAS and 60–90 DAS of soybean crop.

Treatments	SPAD				NDVI				CTD (°C)			
	30–45 DAS		60–90 DAS		30–45 DAS		60–90 DAS		30–45 DAS		60–90 DAS	
	2020	2021	2020	2021	2020	2021	2020	2021	2020	2021	2020	2021
Irrigation												
I_1_	28.2^b^	27.6^b^	33.8^b^	35.2^b^	0.37^c^	0.37^b^	0.53^c^	0.58^b^	3.23^b^	3.70^b^	3.09^c^	3.12^b^
I_2_	30.9^a^	31.7^a^	40.7^a^	42.3^a^	0.48^a^	0.46^a^	0.66^a^	0.67^a^	4.02^a^	4.66^a^	4.14^a^	4.44^a^
I_3_	29.8^ab^	30.2^ab^	37.0^ab^	38.7^b^	0.41^b^	0.41^ab^	0.59^b^	0.63^ab^	3.83^ab^	3.86^ab^	3.53^b^	3.80^ab^
SEm ±	0.34	0.71	0.85	0.92	0.01	0.02	0.01	0.02	0.10	0.08	0.05	0.19
LSD (P=0.05)	1.35	2.80	2.49	3.63	0.03	0.06	0.04	0.06	0.39	0.33	0.18	0.75
Precision nutrient management												
PNM_1_	29.6^b^	31.4^a^	40.2^ab^	40.7^a^	0.43^b^	0.45^ab^	0.61^ab^	0.65^a^	3.83	4.10	3.68	4.03
PNM_2_	29.0^b^	30.5^ab^	36.9^bc^	38.1^b^	0.41^b^	0.41^abc^	0.60^b^	0.63^a^	3.72	4.04	3.49	3.50
PNM_3_	31.0^a^	31.8^a^	42.8^a^	41.9^a^	0.47^a^	0.46^a^	0.62^a^	0.65^a^	3.91	4.15	3.83	4.14
PNM_4_	29.0^b^	28.9^b^	34.5^cd^	37.6^b^	0.40^bc^	0.40^bc^	0.59^b^	0.63^a^	3.58	4.01	3.53	3.72
PNM_5_	28.9^b^	26.6^c^	31.3^d^	35.3^c^	0.38^c^	0.36^c^	0.55^c^	0.56^b^	3.43	4.07	3.41	3.54
SEm ±	0.30	0.51	0.85	0.37	0.01	0.01	0.01	0.01	0.12	0.09	0.11	0.18
LSD (***p*** =0.05)	0.87	1.50	2.49	1.09	0.02	0.04	0.02	0.03	NS	NS	NS	NS
I×PNM	S	S	S	S	S	S	S	S	NS	NS	NS	NS
*Control vs. SCI*												
Control	27.3^b^	27.7^b^	36.3^a^	37.6^b^	0.37^b^	0.42^a^	0.52^b^	0.50^b^	3.27^b^	3.46^b^	2.77^b^	3.45^b^
SCI	29.6^a^	29.8^a^	37.1^a^	38.7^a^	0.42^a^	0.42^a^	0.59^a^	0.63^a^	3.69^a^	4.07^a^	3.59^a^	3.79^a^
SEm ±	0.13	0.23	0.38	0.17	0.004	0.006	0.003	0.004	0.05	0.04	0.05	0.08
LSD (***p*** =0.05)	0.39	0.67	NS	0.49	0.010	NS	0.01	0.013	0.16	0.11	0.14	0.24

I_1_, standard flood irrigation 50% DASM (FI); I_2_, sprinkler irrigation at 80% ET_C_ (Spr 80% ETc); I_3_, sprinkler irrigation at 60% ET_C_ (Spr 60% ETc). For a detailed description of PNM_1–5_, refer to **Table 2**. Values with different superscript letters in a column are significantly (p<0.05) different as determined by Tukey’s honest significant difference test. NS-non significant, whereas S indicates Significant at (p<0.05).

**Figure 4:**
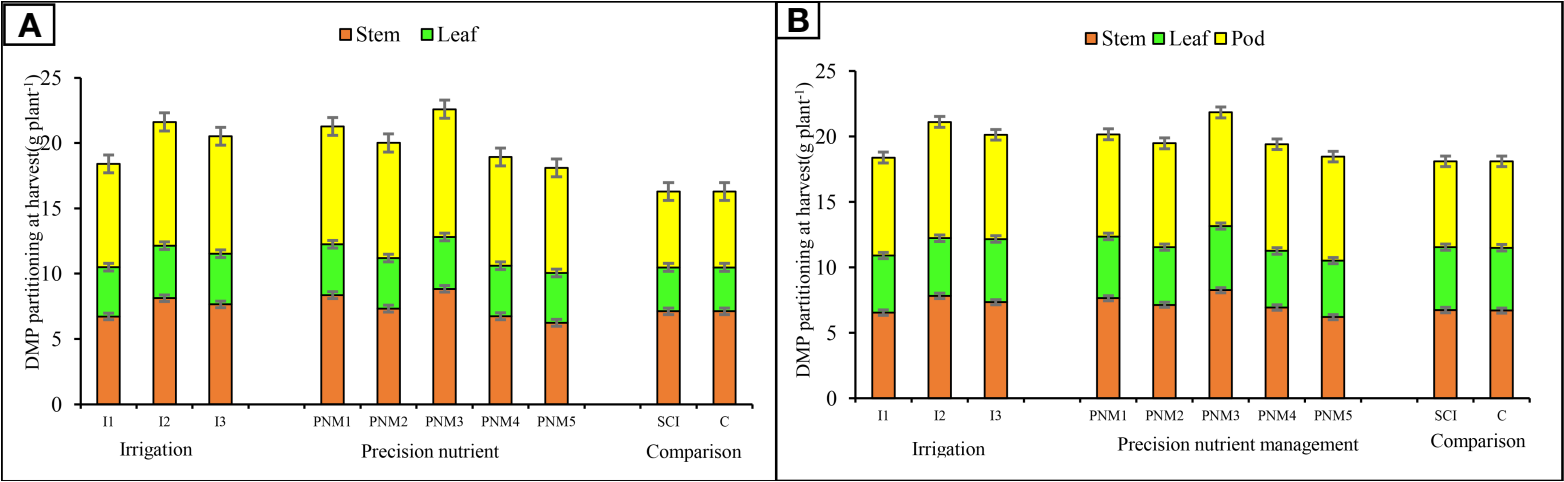
Effect of precision nutrient and irrigation management on dry matter partitioning of soybean (stem, leaves and pods) at harvest 2020 **(A)** and 2021 **(B)**. For a detailed description of I (irrigation) and PNM (precision nutrient management), refer to **Table 2**. Any treatment difference that exceeds the range of the bar within a given year is significantly different, as indicated by the LSD 0.05 bar above each column.

The higher crop growth indices (CGR, RGR, and LAI), dry matter accumulation (DMA), and dry matter partitioning (DMP) of soybean were recorded in Spr 80% ETc, which was followed by Spr 60% ETc and FI during study period (**Table 7**). Spr 80% ETc exhibited significantly (*p*=0.05) higher CGR (8.33 and 8.42 g m^−2^ day) and RGR (77.28 and 66.70 mg g^−1^ day^−1^) over FI during 2020 and 2021. Likewise, plots with Spr 80% Etc irrigation recorded significantly higher LAI at 60 DAS with increments ranging from 26.2% to 28.3% and 29.4% and 43.1% during 2020 and 2021, respectively. The DMA at harvest of soybean also followed a similar trend in both years. The percent increase in stem dry weight (SDW) for Spr 80% ETc was 19.5%–20.7% and 6.1%–6.7% (mean of 2 years) as compared to FI and Spr 60% ETc, respectively (**Figure 4**). Pod dry weight (PDW) increased by 18.7%–19.7% and 11.5%–5.3% than FI and Spr 60% ETc in 2020 and 2021, respectively. No significant difference was observed in LDW (leaf dry weight) in both years.

**Table 7:** Effect of precision nutrient and irrigation management on crop growth rate (CGR), relative growth rate (RGR), leaf area index (LAI), and dry matter accumulation (DMA) in soybean.

Treatments	CGR (g m^−2^ day^−1^)		RGR (mg g^−1^ day^−1^)		LAI		DMA (g m^−2^)	
	2020	2021	2020	2021	2020	2021	2020	2021
Irrigation								
I_1_	7.26^b^	7.54^b^	59.68^b^	51.33^c^	1.93^b^	2.68^b^	228.94^b^	194.17^b^
I_2_	8.33^a^	8.42^a^	77.28^a^	66.70^a^	2.38^a^	3.25^a^	259.14^a^	219.59^a^
I_3_	7.70^ab^	8.14^ab^	67.67^ab^	59.58^b^	2.23^a^	2.96^ab^	245.10^ab^	203.78^ab^
SEm ±	0.17	0.15	2.29	1.22	0.05	0.07	4.73	4.69
LSD (***p*** =0.05)	0.65	0.60	8.99	4.79	0.20	0.29	7.43	18.42
Precision nutrient management								
PNM_1_	7.84^ab^	8.07^b^	69.76^ab^	61.49^ab^	2.27^a^	2.99^ab^	247.21^ab^	210.89^ab^
PNM_2_	7.72^ab^	7.93^bc^	66.35^ab^	59.55^ab^	2.15^ab^	2.95^ab^	243.73^ab^	208.47^ab^
PNM_3_	8.13^a^	8.41^a^	72.63^a^	61.92^a^	2.27^a^	3.13^a^	254.26_a_	216.67^a^
PNM_4_	7.63^ab^	7.91^bc^	67.86^ab^	58.75^b^	2.13^ab^	2.92^b^	239.87^b^	200.16^bc^
PNM_5_	7.50^b^	7.84^c^	64.43^b^	54.30^c^	2.08^b^	2.82^b^	236.91^b^	193.04^c^
SEm ±	0.13	0.08	1.81	1.32	0.05	0.04	3.44	3.60
LSD (***p*** =0.05)	0.38	0.24	5.30	3.85	0.13	0.13	10.06	10.54
I×PNM	NS	NS	NS	NS	NS	NS	NS	NS
*Control vs. SCI*								
Control	7.15^b^	7.48^b^	52.30^b^	57.00^b^	2.45^a^	3.02^a^	278.51^a^	224.17^a^
SCI	7.77^a^	8.03^a^	68.21^a^	59.20^a^	2.18^a^	2.96^a^	244.39^a^	205.84^a^
SEm ±	0.06	0.04	0.81	0.59	0.02	0.02	1.54	1.61
LSD (***p*** =0.05)	0.23	0.11	2.37	1.72	0.06	0.06	NS	NS

I_1_, standard flood irrigation 50% DASM (FI); I_2_, sprinkler irrigation at 80% ET_C_ (Spr 80% ETc); I_3_, sprinkler irrigation at 60% ET_C_ (Spr 60% ETc). For a detailed description of PNM_1–5_, refer to **Table 2**. Values with different superscript letters in a column are significantly (p<0.05) different as determined by Tukey’s honest significant difference test. NS-non significant, whereas S -Significant at (p<0.05).

Significantly higher grain yields of 2.45 t ha^–1^ and 2.56 t ha^–1^ were recorded with the application of Spr 80% ETc than FI, and it was at par with Spr 60% ETc (2.15 t ha^–1^ and 2.30 t ha^–1^). The percent increase in grain yield ranged from 23.1% to 29.1% under Spr 80% ETc over FI plots. A significantly lower grain yield was recorded in FI (1.97 t ha^–1^ and 1.99 t ha^–1^) in both years (**Supplementary T** **able S9**). The highest soybean biological yield was recorded in the Spr 80% ETc (7.61 t ha^–1^ and 7.72 t ha^–1^), which was 13.7%–18.5% higher than FI and 16.0%–20.0% higher than Spr 60% ETc. The FI recorded the least soybean biological yield among the irrigation management practices (6.69 t ha^–1^ and 6.51 t ha^–1^) in both years (**Supplementary T** **able S9**). The grain and biological yield under various irrigation treatments followed the Spr 80% ETc > Spr 60% ETc> FI. Similarly, IWP followed the trend as Spr 60% ETc > Spr 80% ETc > FI across years. Soybean, crop under Spr 60% ETc resulted in highest IWP (0.95; 1.21 kg ha^−1^ m^−3^), whereas Spr 80% ETc recorded the highest EWP (24.8; 18.1 INR ha^−1^ m^−3^) and WUE (7.1; 8.1 kg ha^–1^ mm^–1^ day^−1^), respectively, during 2020 and 2021 (**Table 8**). Likewise, the Spr 80 ETc showed an increment IWP, EWP, and WUE to the extent of 32.6%, 31.08%, and 49.06% over FI (**Table 8**).

**Table 8:** Effect of precision nutrient and irrigation management on total water productivity, irrigation water productivity, and economic water productivity in soybean under SCI.

Treatment	IWP (kg ha^−1^ m^−3^)		EWP (INR ha^–1^ m^−3^)		WUE (kg ha^−1^ mm^−1^ day^−1^)	
	2021	2022	2021	2022	2021	2022
*Irrigation*						
I_1_	0.54^b^	0.66^c^	17.6^c^	13.0^b^	6.2^b^	6.2^c^
I_2_	0.93^a^	1.01^b^	24.8^a^	18.1^a^	7.1^a^	8.1^a^
I_3_	0.95^a^	1.21^a^	23.4^b^	17.8^a^	5.7^c^	7.3^b^
Sem ±	0.017	0.040	0.37	0.56	0.10	0.32
LSD (P=0.05)	0.068	0.155	1.46	2.20	0.39	1.24
*Precision nutrient management*						
PNM_1_	0.82^b^	0.99^ab^	22.6^b^	16.7^ab^	6.5^b^	7.4^ab^
PNM_2_	0.79^bc^	0.97^bc^	21.8^b^	16.4^bc^	6.3^b^	7.3^ab^
PNM_3_	0.89^a^	1.04^a^	24.5^a^	17.5^a^	7.1^a^	7.7^a^
PNM_4_	0.78^bc^	0.88^d^	21.8^b^	15.2^cd^	6.3^b^	6.7^b^
PNM_5_	0.77^c^	0.93^cd^	19.0^c^	15.7^d^	5.4^c^	7.0^ab^
Sem ±	0.012	0.019	0.27	0.35	0.08	0.20
LSD (*p*=0.05)	0.036	0.057	0.78	1.02	0.22	0.60
*Control versus SCI*						
Control	0.46^b^	0.43^b^	15.8^b^	10.8^b^	5.6^b^	5.7^b^
SCI	0.81^a^	0.96^a^	21.9^a^	16.3^a^	6.3^a^	7.2^a^
Sem ±	0.005	0.009	0.12	0.16	0.03	0.09
LSD (*p*=0.05)	0.016	0.025	0.35	0.46	0.10	0.27

I_1_, standard flood irrigation 50% DASM (FI); I_2_, sprinkler irrigation at 80% ET_C_ (Spr 80% ETc); I_3_, sprinkler irrigation at 60% ET_C_ (Spr 60% ETc). For a detailed description of PNM_1–5_, refer to **Table 2**. IWP, irrigation water productivity; EWP, economic water productivity; WUE, water-use efficiency. Values with different superscript letters in a column are significantly (p<0.05) different as determined by Tukey’s honest significant difference test.

### Precision nutrient management effects

Precision nutrient management (PNM) practices exhibited significant (*p*=0.05) improvement in photosynthetic characteristics like Pn, Tr, Gs, Ci, and NAR during 2020 and 2021. The adoption of PNM practices enhanced P_n_ in soybean from 10.36 to 15.90 µmol CO_2_ m^2^ s^−1^ (**Table 5**). Likewise, the adoption of PNM_3_ [point placement of fertilizers and 50% SPAD based N top dressing] exhibited significantly higher P_n_ compared to PNM_5_ with the percent increment ranging from 28.2% and 49% during both study years, respectively. Likewise, soybean leaves under PNM treatments transpired at the rate of 6.22–6.67 m mol H_2_O m^2^ s^−1^ during both years. However, PNM_3_ exhibited 18.9% and 16.1% greater Tr rate than PNM_5_. Similarly, the adoption of PNM_3_ practice enhanced G_S_ by 23.1% and 11.8%, Ci by 14.5% and 7.9%, and NAR by 17.4% and 16.2% over PNM_5_ in both seasons (**Table 5**). However, PNM_2_ and PNM_4_ were statistically comparable across both seasons for most of the photosynthetic parameters. Likewise, PNM practices also enhanced SPAD and NDVI values in soybean. The chlorophyll content and NDVI values in PNM practices followed the order of PNM_3_> PNM_1_>PNM_2_> PNM_4_> PNM_5_ at 30–45 DAS and 60–90 DAS in both seasons (**Table 6**). The SPAD and NDVI values of soybean peaked at 60–90 DAS resulting from SPAD-meter-assisted N top dressing. The SPAD values under PNM ranged from 29 to 38 and 35.3 to 42.8 at 30–45 DAS and 60–90 DAS, respectively, during the study period. NDVI values were significantly higher under PNM_3_ at 30–45 DAS (0.47) and 60–90 DAS (0.64). There was no significant difference in CTD under PNM treatments during any of the study years (**Table 6**), whereas PNM_3_ practice enhanced percent interception of PAR than its PNM_5_ at all the measurement intervals in both years.

The crop growth indices (CGR, RGR, and LAI), DMA, and DMP of soybean were significantly (*p*=0.05) higher under PNM practices during 2020 and 2021 (**Table 7**). The adoption of PNM_3_ recorded significantly higher CGR (7.3% and 3.8% higher), RGR (11.8% and 2.3% higher), and DMA (8.8% and 2.7% higher) over PNM_5_ and PNM_1_ in both the years (**Table 9**). Likewise, superior LAI under PNM_3_ (2.27 and 2.13) was observed than under other PNM treatments. Dry matter fractions of soybean at harvest in both years showed no significant difference in LDW among the PNM treatments (**Figure 4**). However, PNM_3_ showed an increment of 6.5%, 15.3%, and 27.1% over PNM_1,_ PNM_2_, and PNM_5_ in SDW. Likewise, significantly (*p*=0.05) higher PDW was recorded in PNM_3_ over PNM_5_.

**Table 9:** Interaction effects precision nutrient (PNM) and irrigation management (I) on net photosynthetic rate and net assimilation rate of soybean under SCI.

I×PNM	Net photosynthetic rate P_n_ (µmol CO_2_ m^2^ s−^1^)		Net assimilation rate NAR (mg cm^−2^ day^−1^)	
	2020	2021	2020	2021
I_1_×PNM_1_	12.6^defg (ns)^	10.4^gh (*)^	8.7^ef (ns)^	8.7^abc (ns)^
I_1_×PNM_2_	12.6^efg (ns)^	10.9^fg (*)^	10.2^abcde (*)^	8.4^bc (ns)^
I_1_×PNM_3_	13.2^cdefg (ns)^	11.8^efgh (*)^	9.7^bcde (*)^	9.4^abc (ns)^
I_1_×PNM_4_	12.2^efg (ns)^	9.6^h (ns)^	9.6^cde (*)^	8.4^bc (ns)^
I_1_×PNM_5_	11.9^g (ns)^	9.6^h (ns)^	7.8^f (ns)^	6.0^d (ns)^
I_2_×PNM_1_	17.2^a (*)^	15.8^b (*)^	11.0^abc (*)^	10.5^a (*)^
I_2_×PNM_2_	15.5^bcd (*)^	15.1^bc (*)^	10.7^abc (*)^	9.3^abc (ns)^
I_2_×PNM_3_	18.6^a (*)^	19.9^a (*)^	11.8^a (*)^	10.6^a (*)^
I_2_×PNM_4_	15.0^bcdef (*)^	14.9^bc (*)^	9.9^bcde (*)^	9.2^bc (ns)^
I_2_×PNM_5_	12.1^fg (ns)^	9.6^h (ns)^	10.5^abcd (*)^	8.8^abc (ns)^
I_3_×PNM_1_	12.7^defg (ns)^	14.0^bcde (*)^	11.3^ab (*)^	9.2^abc (ns)^
I_3_×PNM_2_	15.0^bcd (*)^	13.3^bcdef (*)^	9.0^def (*)^	8.8^abc (ns)^
I_3_×PNM_3_	15.9^abc (*)^	14.7^bcd (*)^	10.6^abcd (*)^	10.1^ab (*)^
I_3_×PNM_4_	14.1^cdefg (*)^	12.9^cdefg (*)^	10.1^bcde (*)^	8.6^bc (ns)^
I_3_×PNM_5_	13.3^cdefg (ns)^	11.9^defgh (*)^	8.9^def (ns)^	8.0^c^
PNM at same level of I Sem ±	0.57	0.55	0.38	0.37
LSD (0.05)	1.67	1.60	1.12	1.08
I at same or different level of PNM Sem ±	0.63	0.60	0.42	0.39
LSD (0.05)	2.07	1.96	1.36	1.23
*Pairwise comparison of control vs. rest of combination*				
C	13.0	9.4	8.9	9.3
Sem ±	0.15	0.14	0.10	0.09
LSD (***p*** =0.05)	0.43	0.41	0.29	0.25

I_1_, standard flood irrigation 50%DASM (FI); I_2_, sprinkler irrigation at 80% ET_C_ (Spr 80% ET_c_); I_3_, sprinkler irrigation at 60% ET_C_ (Spr 60% ET_c_). For a detailed description of PNM_1–5_, refer **Table 2**. Values with different superscript letters in a column are significantly (p<0.05) different as determined by Tukey’s honest significant difference test. *Parenthesis indicates that I×PNM combination under SCI are significant (p<0.05) over Control, whereas (ns) indicates non-significant difference between C and I×PNM.

The grain yield was significantly (*p*=0.05) higher in PNM_3_ (2.45 t ha^–1^ and 2.43 t ha^–1^) followed by PNM_1_ (2.43 t ha^–1^ and 2.12 t ha^–1^). Likewise, soybean crop under PNM_3_ recorded 7.67 t ha^–1^ and 7.65 t ha^–1^ of biological yield, which was 26.8% and 20.3% higher than soybean biological yield recorded under PNM_5_ in 2020 and 2021, respectively (**Supplementary T** **able S9**). The enhancement in soybean yields under PNM treatments followed the trend PNM_3_>PNM_1_>PNM_2_>PNM_4_>PNM_5_ during the study period. A similar trend with respect to water productivity was observed under PNM practices, where adoption of PNM_3_ recorded significantly higher IWP (0.89 and 1.04 kg ha^−1^ m^−3^), EWP (24.5 and 17.5 INR ha^−1^ m^−3^), and WUE (7.1 and 7.7 kg ha^–1^ mm^–1^ day^−1^) during 2020 and 2021, respectively. The IWP, EWP, and WUE of soybean under PNM_3_ were 15.6%–11.8%, 11.5%–28.9%, and 10.0%–30.1% higher than PNM_5_ during both study years.

### Interaction effects

The photosynthetic characters like net photosynthetic rate (P_n_) and net assimilation rate (NAR) at flowering stage were significantly (*p*=0.05) affected by the interaction effect of irrigation management and PNM practices (**Table 9**). A combination of irrigations and PNM practice enhanced Pn values from 18.61 to 19.97 mol CO_2_ m^2^ s−^1^and NAR values from 6 to 11.8 mg cm^−2^ day^−1^during 2020 and 2021. In both years, the Pn (17.2; 15.8 µmol CO_2_ m^2^ s^−1^) and NAR (11.8; 10.6 mg cm^−2^ day^−1^) of soybean plants under the I_2_×PNM_3_ treatment were higher than other combinations (**Table 10**), whereas the lowest Pn and NAR were recorded with the combination of I_1_×PNM_5_. Likewise, SPAD and NDVI values exhibited significant interaction effect irrigation and PNM practices at 30–45 DAS and 60–90 DAS (**Table 10**). The increase in SPAD due to I_2_×PNM_3_ over I_1_×PNM_5_ varied from 20.97% to 54.7% and 54.3% to 53.2% at 30–45 DAS and 60–90 DAS. Likewise, I_2_×PNM_3_ registered 57%–74% and 52.1%–53.1% increase in NDVI at 30–45 DAS and 60–90 DAS over I_1_×PNM_5_. Pair-wise comparison of individual combination with conventional cultivation showed that Pn, NAR, SPAD, and NDVI were significantly (*p*=0.05) improved by most of the irrigation × PNM combinations over control. Significantly higher grain yield was recorded with the combination of I_2_×PNM_3_ (2.59 and 2.67 t ha^–1^) than other combinations during both study years. The lowest grain yield of 1.7 and 2.01 t ha^–1^ was recorded with I_1_×PNM_5_. Likewise, the highest biological yield was recorded under I_2_×PNM_3_ (8.3 and 8.42 t ha^–1^). The interaction effect of irrigation and PNM practices showed that I_3_×PNM_3_ recorded the highest IWP (0.65 and 0.89 kg ha^−1^ m^−3^), whereas I_2_×PNM_3_ showed highest EWP (29.2 and 33.9 INR ha^–1^ m^3^) and WUE (5.74 and 6.43 kg ha^–1^ mm^–1^ day^−1^) during the study period (**Supplementary T** **able S10**).

**Table 10:** Irrigation management (I) × precision nutrient (PNM) interaction effects on SPAD and NDVI values of soybean under SCI.

I×PNM	SPAD				NDVI			
	30–45 DAS		60–90 DAS		30–45 DAS		60–90 DAS	
	2020	2021	2020	2021	2020	2021	2020	2021
I_1_×PNM_1_	28.3^d (ns)^	29.7^abc (*)^	36.9^bcd (*)^	37.3^efgh (ns)^	0.37^fg (ns)^	0.39^cde (ns)^	0.55^f (*)^	0.61^bc (*)^
I_1_×PNM_2_	28.8^bcd (ns)^	29.3^bc (*)^	33.8^cd (*)^	33.8^h (ns)^	0.35^g (ns)^	0.38^cde (ns)^	0.55^ef (*)^	0.62^bc (*)^
I_1_×PNM_3_	28.5^cd (ns)^	30.95^abc (*)^	41.8^ab (*)^	39.6^cdef (*)^	0.43^cde (*)^	0.45^abc (*)^	0.55^ef (*)^	0.59^c (*)^
I_1_×PNM_4_	28.0^d (ns)^	24.8^de (ns)^	30.9^d (ns)^	35.3^gh (ns)^	0.35^g (ns)^	0.33^de (ns)^	0.54^f (*)^	0.60^bc (*)^
I_1_×PNM_5_	27.4^d (ns)^	23.1^e (ns)^	30.2^d (ns)^	30.0^i (ns)^	0.37^fg (ns)^	0.31^e (ns)^	0.48^g (ns)^	0.47^d (*)^
I_2_×PNM_1_	31.3^ab (*)^	33.4^ab (*)^	41.9^ab (*)^	44.6^a (*)^	0.51^ab (*)^	0.50^ab(ns)^	0.68^ab (*)^	0.69^ab (*)^
I_2_×PNM_2_	29.2^bcd (*)^	31.7^abc (*)^	40.0^abc (*)^	42.5^abc (*)^	0.48^bc (*)^	0.44^abc (*)^	0.64^bcd (*)^	0.65^abc (*)^
I_2_×PNM_3_	33.2^a (*)^	33.9^a (*)^	46.7^a (*)^	46.0^a (*)^	0.55^a (*)^	0.54^a(*)^	0.73^a (*)^	0.72^a (*)^
I_2_×PNM_4_	30.9^abc (*)^	31.2^abc (*)^	40.5^abc (*)^	41.1^bcde (*)^	0.45^cd (*)^	0.45^abc (*)^	0.65^bc (*)^	0.68^abc (*)^
I_2_×PNM_5_	29.9^bcd (ns)^	28.6^cd (*)^	34.3^cd (*)^	39.8^cdef (*)^	0.40^defg (*)^	0.40^bcde (ns)^	0.60^cde (*)^	0.62^bc (*)^
I_3_×PNM_1_	29.3^bcd (ns)^	31.1^abc (*)^	41.4^ab (*)^	41.4^bcd (*)^	0.41^def (*)^	0.45^abc (*)^	0.60^cde (*)^	0.66^abc (*)^
I_3_×PNM_2_	29.1^bcd (ns)^	30.6^abc (*)^	37.0^bcd (ns)^	40.0^defg (ns)^	0.41^def (*)^	0.42^bcde (*)^	0.59^def (*)^	0.63^abc (*)^
I_3_×PNM_3_	31.4^ab (*)^	30.7^abc (*)^	40.0^abc (*)^	41.6^bcd (*)^	0.43^cde (*)^	0.38^cde (*)^	0.60^cdef (*)^	0.63^abc (*)^
I_3_×PNM_4_	30.0^bcd (*)^	30.6^abc (*)^	32.2^d (ns)^	36.3^fgh (ns)^	0.41^def (*)^	0.43^abcd (*)^	0.59^ef (*)^	0.61^bc (*)^
I_3_×PNM_5_	29.3^bcd (ns)^	28.1^cd (*)^	34.2^cd (ns)^	36.1^fgh (ns)^	0.38^(*)^	0.38^cde (ns)^	0.57^ef (*)^	0.60^bc (*)^
PNM at same level of I SEm ±	0.51	0.89	1.47	0.64	0.014	0.022	0.013	0.018
LSD (0.05)	1.50	2.59	4.31	1.88	0.041	0.065	0.039	0.053
I at same or different level of PNM SEm ±	0.57	1.07	1.82	1.09	0.014	0.025	0.016	0.023
LSD (0.05)	1.88	3.60	6.19	3.96	0.046	0.085	0.052	0.079
*Pairwise comparison of control vs. rest of combination*								
C	29.62	27.72	36.30	37.57	0.37	0.42	0.52	0.50
SEm ±	0.13	0.23	0.38	0.15	0.004	0.006	0.003	0.004
LSD (***p*** =0.05)	0.39	0.67	1.11	0.43	0.010	0.017	0.01	0.013

I_1_, standard flood irrigation 50% DASM (FI); I_2_, sprinkler irrigation at 80% ET_C_ (Spr 80% ET_c_); I_3_, sprinkler irrigation at 60% ET_C_ (Spr 60% ET_c_). For a detailed description of PNM_1–5_, refer to **Table 2**. Values with different superscript letters in a column are significantly (p<0.05) different as determined by Tukey’s honest significant difference test. *Parenthesis indicates that I×PNM combination under SCI are significant (p<0.05) over Control, whereas (ns) indicates non-significant difference between C and I×PNM.

### Comparison of SCI versus control

A comparative study of SCI and conventional cultivation of soybean showed that all the photosynthetic characters (Pn, Tr, Gs, and Ci) under study showed significant (*p*=0.05) improvement under SCI over control during both years of study. Likewise, crop growth indices, *viz.*, CGR, RGR, and DMP, also exhibited significant (*p*=0.05) edge over control, whereas growth indices like LAI, DMA, and PAR interception were found to be similar in SCI and conventional practices. A comparison of SCI versus control evidenced significantly higher grain yield (2.20 and 2.28 t ha^–1^) with the percent increase of 13% and 27% in both the cropping season (**Supplementary T** **able S9**). During 2020, there was no significant difference between SCI and conventional for biological, while in the second year, SCI cultivation (6.96 t ha^–1^) produced significantly higher biological yield than conventional cultivation. Water productivity improved significantly (*p*=0.05) under SCI over control.

### Multivariate analysis and principal component analysis

**Figure 5:**
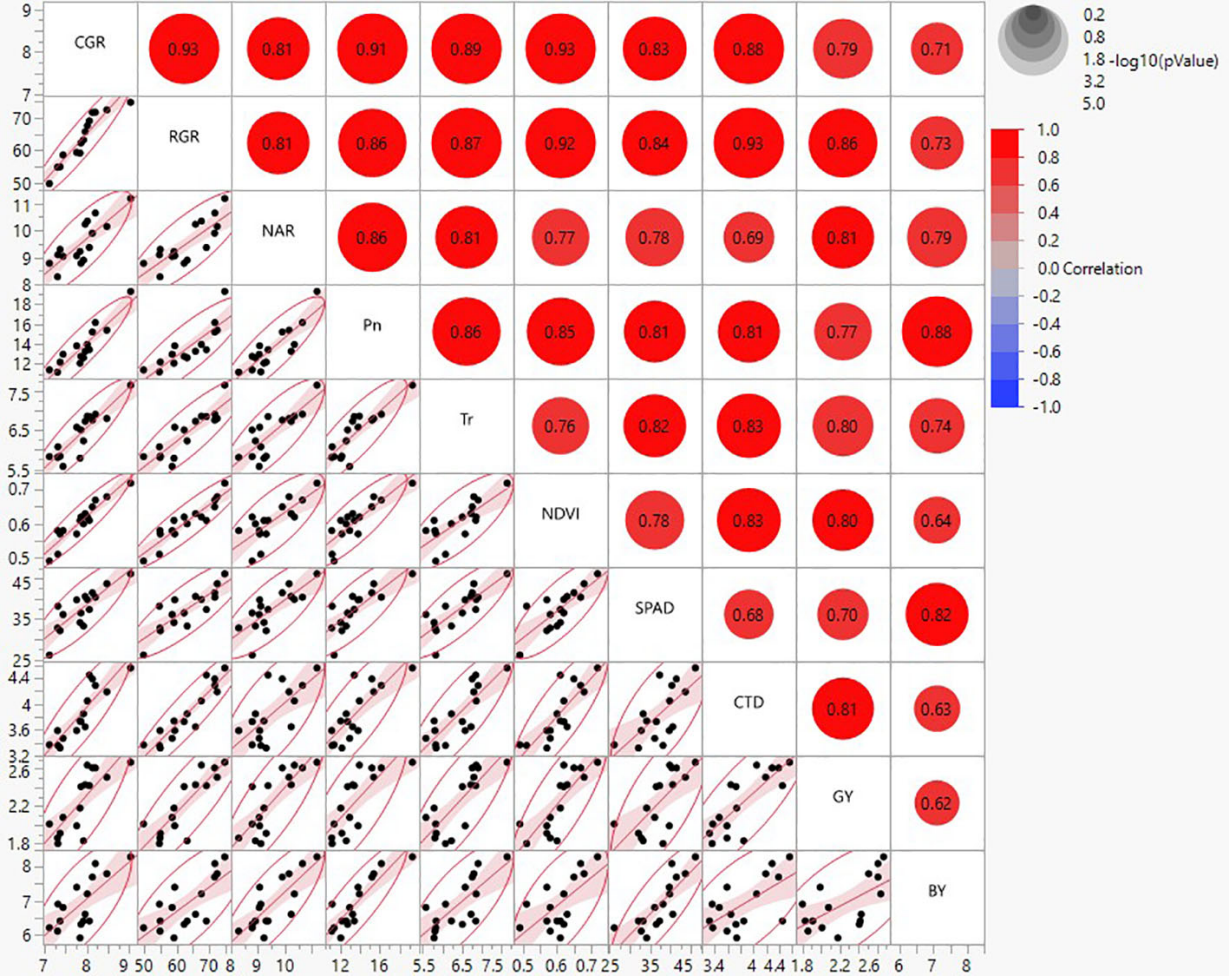
Multivariate analysis showing correlation between various crop growth indices (CGR, RGR, and NAR at 60 DAS), physiological, sensor parameters (Pn, Tr, NDVI, SPAD, and CTD at flowering stage), grain (GY), and biological yield (BY) during 2020 and 2021. The upper triangle displays the significant circles with correlation coefficient (*p*=0.05) (2-year pooled data), while the lower triangle displays the scatter plot matrix with line fit.

**Figure 6:**
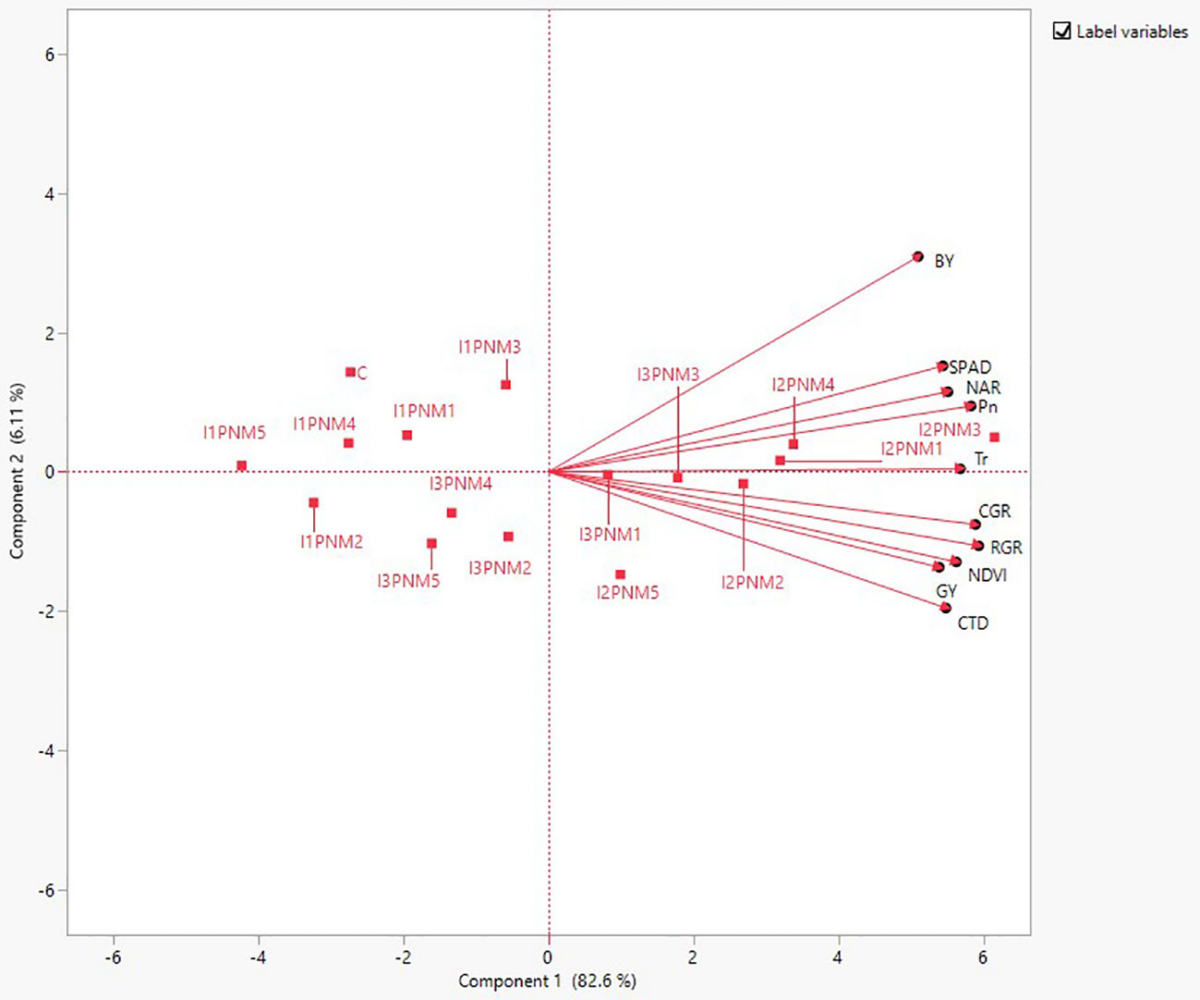
PCA biplots on the effect of precision nutrient and irrigation management on physiological and sensor parameters (Pn, Tr, NAR, SPAD, NDVI, and CTD) on grain yield (GY) and biological yield (BY) of soybean from the pooled data of 2020 and 2021. Squares in red color indicate treatment combinations of I×PNM, and C indicates control.

The association of the relationship between different physiological parameters, crop growth indices, and productivity was studied through multivariate analysis (MVA) (**Figure 5**). Predominantly, most of the physiological and growth indices variables were positively correlated with grain yield (GY) and biological yield (BY) (**Figure 5**). Nevertheless, a strong positive correlation was observed in Pn (r = 0.77 and 0.88), Tr (r=0.80 and 0.74), CTD (r=0.81 and 0.63), and CGR (0.79 and 0.71) for GY and BY, respectively. Furthermore, chlorophyll index (SPAD) and NDVI exhibited a strong association with GY (r = 0.80 and 0.70); however, SPAD showed intense association with BY (r= 0.82), while the NDVI showed stronger association with GY (r=0.80). Dimension reduction in data set and the most important variables contributing yield was obtained by principal component analysis (PCA) (**Figure 6**). PCA showed the superiority of sprinkler irrigation (Spr 80% ETc) and precision nutrient management (PNM) over FI ×PNM combinations. GY is closely related with Tr, Pn, NDVI, CTD, CGR, RGR, and RGR except BY and SPAD, which are found to be less associated.

## Discussion

### Irrigation effect

Among the irrigation management practices, the adoption of sprinkler irrigation at 80% ET_c_ (Spr 80% ETc) resulted in an increment of 25.6%, 17.6%, 35.4%, and 17.5% in P_n_, T_r_, G_s_, and C_i_ compared to standard flood irrigation at 50% DASM (FI). Sprinkler irrigation alters the crop’ s microclimate by lowering canopy temperature (**Table 7**) and maintaining plant water status (Li et al., 2013; Bana et al., 2020; Liu et al., 2021; Tang et al., 2022) and eventually leads to better net-photosynthetic rate in soybean (Cotrim et al., 2021; Rajanna et al., 2022b; Wang et al., 2023). Additionally, Pinnamaneni et al. (2022) observed that soybean cultivated in a rainfed environment or without irrigation exhibited a decrease in stomatal conductance and transpiration, which in turn decreased the crop’s ability to perform photosynthetically. The enhancement in SPAD and NDVI values under sprinkler irrigation could be attributed to sufficient availability of moisture, and the solubility of nutrients helped the crops to grow luxuriantly, which was reflected in higher SPAD and NDVI values at various growth stages (**Supplementary Tables S3–S6**).

The improved photosynthetic characters and chlorophyll content of the plant helps in better assimilation of nutrients from the soil, thereby improving the crop growth indices (**Table 7**). The improved crop growth indices and DMA in Spr 80% Etc and Spr 60% ETc irrigation applied plots could be attributed to optimum soil profile moisture and good aeration throughout the effective root zone of the crop (Bhattarai et al., 2004; Daisy et al., 2013; He et al., 2017; Montoya et al., 2017). Earlier studies show that under deficit irrigation or moisture stress condition crop growth get hampered (Xu et al., 2016; Ahmed et al., 2019; Birthal et al., 2021). The adoption of ET_c_-based irrigation application in soybean has a direct relationship with transpiration, where the greater the ET_c_, the more the transpiration, which, in turn, enhances the photosynthesis through better exchange of gases and greater chlorophyll content. Therefore, sprinkler irrigation based on ET_c_ (Spr 80% ETc and Spr 60% ETC) resulted in improved crop growth and development (Sincik et al., 2008; Irmak et al., 2013; Anda et al., 2020; Da Silva et al., 2022; Sandhu and Irmak, 2022; Tavares et al., 2022). Moreover, it reduces the amount of nutrients that are lost through leaching because irrigation water was applied depending on the ETc of the crop through sprinklers, avoiding an excessive application as opposed to normal flood irrigation (**Table 4**). Yang et al. (2020) showed that sprinkler irrigation reduced the nitrate leaching loss (44%) below the root zone, which is attributed to the fact that the wetting front is shallow under sprinkler irrigation compared to furrow irrigation. Application of excess water more than the crop needs resulted in deep percolation in FI leading to leaching loss of applied nutrients along with water (Katoh et al., 2004; Alva et al., 2006; Zhou et al., 2006). Irrigation of crop with Spr 80% ETc and Spr 60% ETc practice resulted in better photosynthates partitioning toward sink (pod dry weight) over FI (**Figure 4**). The positive correlation between CGR, NAR, and DMA with grain yield showed positive impact of sprinkler irrigation on yield of soybean (**Figure 5**). The lowest grain yield was recorded in FI (1.97 t ha^−1^ and 1.99 t ha^−1^); even though the crop under FI received higher amount of irrigation water, the greater part of it might not be effectively utilized by the crop for its metabolic process because of deeper percolation loss of applied irrigation water beyond the effective root zone under the influence of gravity (Abbasi et al., 2003). The higher water requirements coupled with greater evapo-transpiration under FI resulted in less water productivity (**Table 8**). Ghazanfar et al. (2010) explained that the significant increase in applied water under FI could be due to the higher evaporation during the growing period, while the sprinkler irrigation minimizes evaporation losses from the soil and from crop canopy surface and also applying irrigation based on crop water needs (ETc) improved WUE of soybean under Spr 60% ETc (Kang et al., 2014; Gajic et al., 2018; Uddin and Murphy, 2020; Djaman et al., 2022). The irrigation management practices had greatly influenced the yield of the soybean crop in our current study through enhanced physiological process (net photosynthesis and transpiration) and resource-use efficiency (water productivity and water-use efficiency).

### PNM effect

Basal point placement of fertilizer coupled with top dressing of remaining 50% N fertilizer with SPAD-based management (PNM_3_) exhibited significantly higher P_n_, T_r_, G_s_, and C_i_ over PNM_5_ (**Table 5**). Therefore, the point placement of fertilizer around the root zone of the crop and need-based application of N fertilizer through SPAD-guided N top dressing helps in maximum acquisition of applied nutrients by the crop. This resulted in quick canopy development by interception of maximum photosynthetically active radiation (PAR) (Hergert et al., 2012; Nkebiwe et al., 2016; Bechtaoui et al., 2021). Higher PAR interception (**Figures 3A, B**) corroborates longer photosynthetic duration resulting in improved photosynthetic characters like Pn, T_r_, G_s_, and C_i_ (Richards, 2000; Thakur et al., 2010; Zhu et al., 2010; Bhagat et al., 2017; Elicin et al., 2021). Likewise, an increment of 29.2% in chlorophyll content (SPAD) at 60–90 DAS was recorded under PNM treatments after SPAD-assisted top dressing of N (**Table 6**). The increase in chlorophyll content is attributed to the advancement in crop age, as there is more chlorophyll biosynthesis due to greater leaf size of the plant (Buttery and Buzzell, 1977; Costa et al., 2001; Pramanik and Bera, 2013; Islam et al., 2014; Kandel, 2020). Therefore, nitrogen application by using a SPAD meter would help save nitrogen fertilizer and enhance the growth and yield of different crops (Behera et al., 2007; Islam et al., 2014; Ali, 2020; Dass et al., 2022; Rajanna et al., 2022a). Thus, enhancement in NDVI values at 60–90 DAS, which has a positive correlation (*r*=0.78) with SPAD (**Figure 5**, **Table 7**), shows that sensor-based PNM helps in improving chlorophyll content of the plant.

PNM practices also help in enhancing crop growth indices (CGR, RGR, NAR, and LAI) through better acquisition of nutrients (**Table 7**). Siyal et al. (2012) found that the placement of fertilizer in the root zone of crop reduces nitrogen leaching from 0% to 33% over surface application. Likewise, point placement of fertilizer resulted in improved nutrient-use efficiency, since higher concentration of nutrients around the root zone results in better uptake of nutrients and minimizes losses (Schmitt et al., 2001; Fengqin et al., 2018; Dass et al., 2019; Nayak et al., 2022; Paramesh et al., 2023). Similarly, in our current study, point placement of fertilizer (PNM_3_) performed significantly (*p*=0.05) superior in nutrient concentration as against PNM_2_, PNM_4_, and PNM_5_, since the nutrient forage area of roots in PNM_2,4,5_ is reduced, resulting in suboptimal crop growth (CGR, RGR, and NAR) and DMA. Furthermore, frequent hoeing and PNM_1_ (SCI nutrient management), which combined organic and inorganic nutrient management practices, led to better root growth, which might have increased nutrient uptake when fertilizer is applied in specific locations (Singh et al., 2018; Dass et al., 2019; Parihar et al., 2020). Point placement of fertilizer coupled with SPAD-assisted N management resulted in higher resource-use efficiency with minimum wastage of resource, which corroborated the improvement in yield of soybean (Dass et al., 2014; Dass et al., 2019; Rajanna et al., 2022a). Optimum resource-use efficiency augmented higher water productivity that, in turn, enhanced crop yields with less water requirement (Burt et al., 2005; Liu et al., 2011; Jha et al., 2019). Precise application of nutrients around the root zone of crop resulted in optimum crop growth and development and enabled the plant roots to extract moisture from deeper layers, which ultimately enhanced crop water productivity, *viz*., IWP, EWP, and WUE (Mishra and Salokhe, 2011; Parihar et al., 2017; Jat et al., 2018; Sadhukhan et al., 2023). The precise nutrient management practices had greatly influenced the yield of the soybean crop in our current study through enhanced physiological process (net photosynthesis and transpiration) and resource-use efficiency, *viz.*, water productivity and nutrient-use efficiency through site-specific application.

### Irrigation and PNM Interaction effect

**Figure 7:**
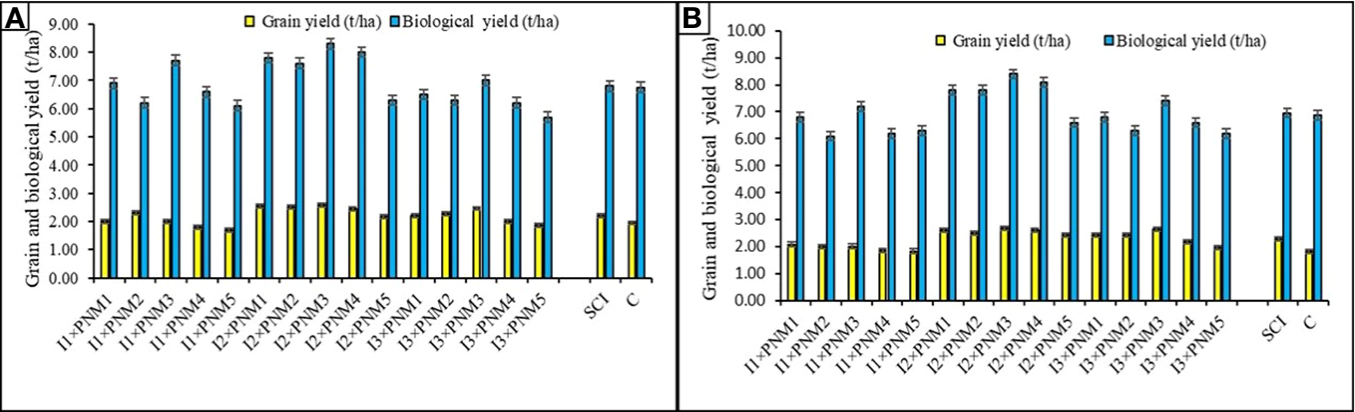
Irrigation management (I) × precision nutrient (PNM) interaction effects on grain yield and biological yield of soybean under SCI 2020 **(A)** and 2021 **(B)**. For a detailed description of I (irrigation) and PNM (precision nutrient management), refer to **Table 2**. Any treatment difference that exceeds the range of the bar within a given year is significantly different, as indicated by the LSD 0.05 bar above each column.

The significant interaction effect of P_n_, NAR, SPAD, and NDVI reiterates the positive effect of I ×PNM on crop growth performances (**Table 9**, **Table 10**). Addendum of all the above sensor based precision nutrient and irrigation management helps in better performance of crop under SCI. Prior studies also showed similar results regarding SPAD, NDVI, and chlorophyll content in improving the performance of crop under sensor-based management system (Raun et al., 2005; Bandyopadhyay et al., 2010; Mishra and Salokhe, 2011; Baral et al., 2021; Rajanna et al., 2022a; Farias et al., 2023). The interaction effect on grain and biological yield corroborates that the integration of I and PNM practice along with SCI significantly helped in improving the grain and biological yield of soybean (**Figure 7**). The application of nutrients and irrigation water as per the needs of the crop through sensor-guided tools, *viz.*, SPAD, NDVI, infrared thermometer, and moisture meter helps in significantly higher yield of crop with minimum wastage of resources (Ma et al., 1995; Sapkota et al., 2014; Shah et al., 2021; Varinderpal et al., 2021).

### SCI versus conventional cultivation

The cultivation of soybean under SCI improved photosynthetic characters in both years. Similarly, in rice under SRI system, wider spacing resulted in better root development, which resulted in higher transportation and accumulation of N and cytokinin from root to shoot, thereby increasing the P_n_ of the rice plant (Stoop et al., 2002; Dass and Chandra, 2013). Similarly, in our current study, soybean under SCI showed improved photosynthetic characters over control. SCI exhibited increment of 7.4%, 13.5%, 4.3%, and 13.7% in CGR, RGR, NAR, and LAI, respectively (**Table 7**), which could be attributed to reduced plant to plant competition for nutrient, moisture, and better interception of PAR (Bullock et al., 1988; Stoop et al., 2002; Singh et al., 2023). However, DMA and PAR interception (**Figures 3A, B**) were higher in conventional cultivation (control) plots compared to SCI cultivation, since plant population per unit area is higher under control, which significantly (*p*=0.05) contributed to DMA (**Table 7**), whereas under SCI, wider spacing (30×30 cm) resulted in lower DMA (Abraham et al., 2014). A reduction in interplant competition under SCI cultivation resulting from optimum crop geometry contributes significantly higher photosynthates partitioning toward sink (Kumar et al., 2011; Divya et al., 2015; Nayaka et al., 2021). SCI practices have positive impact on growth and yield of crops by providing optimum space for individual plant to exploit the native available resources (Dobermann, 2004; Kuttamani and Velayudham, 2016; Dass et al., 2023) coupled with better interception of light and improved acquisition of moisture and nutrients (Singh et al., 2018). Thus, sensor-based precision nutrient and irrigation management under SCI favored the physiology, growth, and development of soybean with improved utilization of resources that ultimately enhances crop productivity. Crop management practices also affect the irrigation water requirement through water consumption by changing soil permeability, and evaporation water consumption of plants; likewise, repeated inter-cultivation under SCI resulted in conserving soil moisture by acting as soil mulch (Singh et al., 2022).

### Limitation of the present study

The limitation of the current study offers the scope for the development of machine that will carry out both sowing and fertilizer placement simultaneously, which in turn reduces the sowing and fertilizer placement cost of labor. The lack of proper regulation over the sprinkler system, since it is portable, and the measurement of photosynthetic parameters at regular intervals of crop phenology will help in better understanding and give better insights how sensor tools help in providing favorable microclimate for crop growth.

## Conclusion

The current work provides valuable insights into the mechanisms by which sensor-guided instruments enable precise water and nutrition utilization in soybean crop intensification (SCI). Utilizing SCI enhances the crop productivity through improved physiological and growth parameters. Similarly, the adoption of sprinkler irrigation at 80% ET_C_ (Spr 80% ETc) showed superiority for all the physiological, growth, and yield parameters over sprinkler irrigation at 60% (Spr 60% ET_C_) ET_C_ and FI (standard flood irrigation at 50% DASM).Thus, the application of irrigation at Spr 80% ETc resulted in an increment of 25.6%, 17.6% 35.4%, and 17.5% in photosynthetic rate, transpiration rate, stomatal conductance, and intercellular CO_2_ concentration over flood irrigation plots. Among the PNM, PNM_3_ showed significant improvement in photosynthetic characters, SPAD, NDVI, including grain and stover yield. A comparison of SCI versus control showed that crop productivity was enhanced significantly (*p* = 0.05) under SCI cultivation due to improved photosynthetic characters, growth, and development of crop. The enhancement in the grain yield under SCI was to the tune of 19.7% over control. The trend of performance for irrigation (I) and precision nutrient management (PNM) for photosynthetic characters, SPAD (greenness), NDVI, crop growth indices, and yield was Spr 80% ETc >Spr 60% ET_C_>FI and P_3_>P_1_> P_2_> P_4_>P_5_. However, for water productivity under irrigation, the trend was Spr 60% ETc> Spr 80% ETc>FI. Therefore, using sensor-based precision nutrient and irrigation management technique appears to be a successful strategy for improving crop production and maximizing resource utilization in the cultivation of soybeans across a wide range of geographical regions.

## Data availability statement

The original contributions presented in the study are included in the article/**Supplementary Material**. Further inquiries can be directed to the corresponding author.

## Author contributions

SKS: Conceptualization, Investigation, Methodology, Writing – review & editing, Resources, Writing – original draft. AD: Conceptualization, Investigation, Methodology, Project administration, Supervision, Writing – review & editing. SD: Conceptualization, Methodology, Project administration, Writing – review & editing. RG: Methodology, Writing – review & editing. TS: Writing – original draft. SS: Writing – original draft. MS: Formal analysis, Writing – review & editing. AC: Formal analysis, Writing – review & editing. HK: Data curation, Writing – original draft. PBR: Writing – review & editing. SP: Formal analysis, Writing – review & editing. VKS: Formal analysis, Writing – review & editing. VP: Formal analysis, Writing – review & editing. PK: Data curation, Writing – original draft. MK: Writing – review & editing. RS: Data curation, Writing – original draft. VT: Writing – review & editing. KK: Writing – review & editing. KNK: Data curation, Writing – original draft. A-AS: Data curation, Writing – original draft. ADD: Writing – review & editing.

## Glossary

**Table:**

C_i_	Intercellular CO_2_ concentration
CTD	Canopy temperature depression
DAS	Days after sowing
DASM	Depletion of available soil moisture
DMA	Dry matter accumulation
DMP	Dry matter partitioning
ET_0_	Reference evapotranspiration
ET_c_	Crop Evapotranspiration
EWP	Economic water productivity
g	grams
G_s_	Stomatal conductance
GY	Grain yield
Ha	Hectare
I	Irrigation
IWP	Irrigation water productivity
IRGA	Infrared gas analyzer
K	Potassium
K_2_O	Potassium in oxidized form
MVA	Multivariate analysis
LAI	Leaf area index
LDW	Leaf dry weight
LSD	Least significant difference
P	Phosphorus
P_2_O	Phosphorus in oxidized form
PDW	Pod dry weight
P_n_	Net photosynthetic rate
PAR	Photo synthetically active radiation
PCA	Principal component analysis
PNM	Precision nutrient management
PSB	phosphorus solubilizing bacteria
RDF	Recommended dose of fertilizer
SCI	System of crop intensification
SRI	System of rice intensification
SDW	Stem dry weight
SPAD	Soil plant analysis development
STCR	Soil test-based recommendation
SY	Stover yield
T_r_	Leaf transpiration rate
t ha^−1^	Tons ha^−1^
v/v	Volume/volume
WUE	Water use efficiency
NIR	Near-infrared
NAR	Net assimilation rate
RDF	Recommended dose of fertilizer
N	Nitrogen
