# Corrigendum: Sensor-based precision nutrient and irrigation management enhances the physiological performance, water productivity, and yield of soybean under system of crop intensification

**DOI:** 10.3389/fpls.2024.1389386

**Published:** 2024-04-17

**Authors:** K. S. Sachin, Anchal Dass, Shiva Dhar, G. A. Rajanna, Teekam Singh, Susama Sudhishri, Manjanagouda S. Sannagoudar, Anil K. Choudhary, Hari Lal Kushwaha, B. R. Praveen, Shiv Prasad, Vinod Kumar Sharma, Vijay Pooniya, Prameela Krishnan, Manoj Khanna, Raj Singh, T. Varatharajan, Kavita Kumari, Kadagonda Nithinkumar, Aye-Aye San, Ayekpam Dollina Devi

**Affiliations:** ^1^ ICAR–Indian Agricultural Research Institute, New Delhi, India; ^2^ ICAR-Directorate of Groundnut Research, Regional Station, Ananatpur, Andhra Pradesh, India; ^3^ ICAR-Indian Institute of Seed Science, Regional Station, Bengaluru, India; ^4^ ICAR-Central Potato Research Institute, Shimla, India; ^5^ ICAR-National Dairy Research Institute, Karnal, India; ^6^ ICAR-National Rice Research Institute, Cuttack, India; ^7^ Department of Agricultural Research, Regional Research Centre, Aung Ban, Myanmar

**Keywords:** precision nutrient management, sprinkler irrigation, SPAD, photosynthetic rate, PAR interception, SCI, water productivity, soybean yield

In the published article, there was an error in **Discussion**, *Irrigation and PNM Interaction effect*, 5^th^ Paragraph. Only “Table 10” was cited instead of both “Table 9” and “Table 10”; “Gosal et al., 2021” was cited instead of “Varinderpal et al., 2021.”

A correction has been made to **Discussion**, *Irrigation and PNM Interaction effect*, 5^th^ Paragraph. This sentence previously stated:

“The significant interaction effect of P_n_, SPAD, and NDVI reiterates the positive effect of I ×PNM on crop growth performances (Table 10). Addendum of all the above sensor-based precision nutrient and irrigation management helps in better performance of crop under SCI. Prior studies also showed similar results regarding SPAD, NDVI, and chlorophyll content in improving the performance of crop under sensor-based management system (Raun et al., 2005; Bandyopadhyay et al., 2010; Mishra and Salokhe, 2011; Baral et al., 2021; Rajanna et al., 2022a; Farias et al., 2023). The interaction effect on grain and biological yield corroborates that the integration of I and PNM practice along with SCI significantly helped in improving the grain and biological yield of soybean (Figure 7). The application of nutrients and irrigation water as per the needs of the crop through sensor-guided tools, *viz*., SPAD, NDVI, infrared thermometer, and moisture meter helps in significantly higher yield of crop with minimum wastage of resources (Ma et al., 1995; Sapkota et al., 2014; Gosal et al., 2021; Shah et al., 2021).”

The corrected sentence appears below:

“The significant interaction effect of P_n_, NAR, SPAD, and NDVI reiterates the positive effect of I ×PNM on crop growth performances (Table 9, Table 10). Addendum of all the above sensor based precision nutrient and irrigation management helps in better performance of crop under SCI. Prior studies also showed similar results regarding SPAD, NDVI, and chlorophyll content in improving the performance of crop under sensor-based management system (Raun et al., 2005; Bandyopadhyay et al., 2010; Mishra and Salokhe, 2011; Baral et al., 2021; Rajanna et al., 2022a; Farias et al., 2023). The interaction effect on grain and biological yield corroborates that the integration of I and PNM practice along with SCI significantly helped in improving the grain and biological yield of soybean (Figure 7). The application of nutrients and irrigation water as per the needs of the crop through sensor-guided tools, *viz*., SPAD, NDVI, infrared thermometer, and moisture meter helps in significantly higher yield of crop with minimum wastage of resources (Ma et al., 1995; Sapkota et al., 2014; Shah et al., 2021; Varinderpal et al., 2021).”

In the published article, the reference for “Varinderpal et al., 2021” was incorrectly written as “Gosal, S. K., Choudhary, R., Singh, R., and Adholeya, A. (2021). Improving nitrogen use efficiency using precision nitrogen management in wheat (*Triticum aestivum* L.). *J. Plant. Nutr. Soil Sci*. 184 (3), 371–377. doi: 10.1002/jpln.202000371”. It should be “Varinderpal, S., Kunal, Gosal, S. K., Choudhary, R., Singh, R., and Adholeya, A. (2021). Improving nitrogen use efficiency using precision nitrogen management in wheat (*Triticum aestivum* L.). *J. Plant. Nutr. Soil Sci*. 184 (3), 371–377. doi: 10.1002/jpln.202000371”.

The authors apologize for these errors and state that this does not change the scientific conclusions of the article in any way. The original article has been updated.

