# Supplementary material for: Sensor-based precision nutrient and irrigation management enhances the physiological performance, water productivity, and yield of soybean under system of crop intensification
Source: Front Plant Sci. 2023 Dec 18;14:1282217. doi: 10.3389/fpls.2023.1282217 (PMC10773766; doi:10.3389/fpls.2023.1282217)
Supplement: Supplementary file 1 [file DataSheet_1.pdf]

Table1 weekly weather parameters during the *Kharif* 2020

Standard meteorological weeks	Max. Temp	Min. Temp	Rainfall	Wind Speed	RH-max	RH-min	sunshine hours	Evaporation
27	37.0	27.0	18.8	6.5	77.6	57.3	5.1	5.5
28	36.9	27.0	171.8	6.4	83.1	52.0	7.0	5.5
29	34.5	26.4	34.2	5.6	88.7	73.0	2.7	4.7
30	34.0	27.2	3.4	4.4	86.7	63.9	6.4	5.2
31	34.6	28.0	14.8	4.5	85.7	69.3	5.6	5.8
32	35.8	27.6	203.2	7.0	84.9	66.7	4.5	5.5
33	33.4	26.6	68.1	3.8	93.1	79.9	3.3	3.7
34	31.7	25.8	63.2	5.5	89.0	70.6	4.5	3.9
35	33.1	26.3	9.8	4.9	87.4	68.9	5.2	4.0
36	34.5	25.6	0.0	3.3	90.0	66.0	5.8	2.9
37	36.1	23.5	0.0	3.4	89.3	57.6	7.2	4.8
38	37.1	24.9	0.0	4.4	84.3	51.6	7.8	5.3
39	35.4	22.8	0.0	4.1	78.7	57.0	7.5	5.1
40	35.4	19.8	0.0	4.3	76.1	45.7	8.9	6.0
41	34.9	19.8	0.0	2.6	81.9	41.6	7.3	5.3
42	34.1	16.2	0.0	2.0	82.3	32.0	6.7	4.3
43	32.8	12.9	0.0	1.9	85.7	31.4	5.5	3.5
44	30.2	10.9	0.0	2.2	86.6	28.7	4.8	3.0
45	28.7	10.5	0.0	1.6	86.4	32.0	1.6	2.8

[Source: Agro-meteorological Observatory, Division of Agricultural Physics, ICAR-IARI, New Delhi].

Table 2 Weekly weather parameters during the *kharif* 2021

Standard meteorological weeks	Max. Temp	Min. Temp	Rainfall	Wind Speed	RH-max	RH-min	sunshine hours	Evaporation
27	40.4	28.7	95.5	6.8	76.1	45.9	7.7	7.4
28	36.9	27.2	198.6	7.2	85.0	67.1	4.8	5.8
29	34.2	26.5	155.0	3.8	88.0	74.6	3.8	4.1
30	32.8	26.8	66.4	3.5	90.7	81.4	3.0	3.1
31	32.6	25.8	79.6	3.1	90.7	79.4	3.6	3.4
32	34.1	26.4	0.0	4.5	89.3	71.0	3.8	3.5
33	36.5	27.8	128.0	5.9	83.6	61.1	6.7	5.7
34	34.2	26.6	277.2	5.1	87.4	66.3	3.8	3.7
35	32.9	25.3	57.6	3.9	91.0	80.4	3.0	3.4
36	32.9	25.4	170.8	2.8	92.3	80.3	4.5	3.0
37	32.2	24.5	23.0	4.0	91.4	80.0	4.5	3.1
38	32.3	24.7	0.0	3.7	87.7	74.1	5.0	3.8
39	33.7	25.0	36.0	3.4	88.4	70.6	5.6	3.7
40	34.5	24.6	0.0	2.6	90.6	66.0	7.6	3.9
41	35.0	20.5	54.7	2.9	88.3	48.1	8.7	4.3
42	29.7	18.7	37.0	3.4	88.4	63.1	5.8	3.5
43	30.1	15.9	0.0	3.0	86.6	61.4	8.1	3.4
44	29.4	13.6	0.0	2.0	92.6	55.1	7.2	3.4
45	28.1	12.0	0.0	1.7	93.4	50.3	2.8	2.4

[Source: Agro-meteorological Observatory, Division of Agricultural Physics, ICAR-IARI, New Delhi].

Table 3 SPAD (Chlorophyll)at weekly intervals between 30-45 DAS and 60-90 DAS during 2020

	30-45 DAS			60-90 DAS		
**Treatments**	**1st week**	**2nd week**	**3rd week**	**1st week**	**2nd week**	**3rd week**
$I_1 \times PNM_1$	27.25	28.47	29.13	36.25	35.20	39.27
$I_1 \times PNM_2$	26.87	29.46	30.13	32.34	30.50	38.58
$I_1 \times PNM_3$	27.80	29.32	28.25	38.20	43.02	44.31
$I_1 \times PNM_4$	26.87	28.46	28.63	28.25	33.25	31.25
$I_1 \times PNM_5$	26.06	27.20	28.98	22.50	27.25	26.50
$I_2 \times PNM_1$	31.20	32.49	30.20	39.16	39.26	47.16
$I_2 \times PNM_2$	28.50	29.50	29.50	38.20	41.60	40.20
$I_2 \times PNM_3$	33.68	32.20	33.60	48.25	47.25	44.50
$I_2 \times PNM_4$	30.70	30.31	31.79	41.15	40.15	40.25
$I_2 \times PNM_5$	30.00	29.51	30.25	31.50	37.01	34.50
$I_3 \times PNM_1$	29.50	29.25	29.01	39.50	45.20	40.50
$I_3 \times PNM_2$	29.50	28.50	29.25	33.50	40.50	37.02
$I_3 \times PNM_3$	32.50	31.50	30.25	37.61	42.20	40.25
$I_3 \times PNM_4$	29.75	29.26	30.89	28.00	37.00	31.50
$I_3 \times PNM_5$	27.80	29.80	30.25	31.50	41.50	29.50
Control	26.25	27.25	28.49	36.50	38.20	34.19

For detailed description of I (Irrigation) and PNM (Precision nutrient management) refer main table 2

Table 4 SPAD (Chlorophyll) at weekly intervals between 30-45 DAS and 60-90 DAS during 2021

	30-45 DAS			60-90 DAS		
**Treatments**	**1st week**	**2nd week**	**3rd week**	**1st week**	**2nd week**	**3rd week**
$I_1 \times PNM_1$	27.24	28.25	33.56	37.08	35.77	39.05
$I_1 \times PNM_2$	28.25	29.25	30.44	33.38	32.71	35.16
$I_1 \times PNM_3$	31.15	31.20	30.49	39.18	38.49	41.06
$I_1 \times PNM_4$	22.92	25.25	26.25	35.28	33.41	37.15
$I_1 \times PNM_5$	21.96	23.75	23.45	28.78	28.96	32.25
$I_2 \times PNM_1$	34.38	32.80	32.92	42.28	43.25	44.25
$I_2 \times PNM_2$	31.09	31.24	32.67	42.15	42.13	43.12
$I_2 \times PNM_3$	35.20	32.20	34.20	44.15	46.15	43.52
$I_2 \times PNM_4$	30.90	31.50	31.25	40.12	43.10	40.20
$I_2 \times PNM_5$	28.28	29.25	28.25	39.15	40.15	40.25
$I_3 \times PNM_1$	28.28	30.80	34.21	42.49	41.22	40.46
$I_3 \times PNM_2$	29.81	28.94	32.95	38.25	40.36	35.25
$I_3 \times PNM_3$	27.15	30.96	33.88	42.39	42.19	40.14
$I_3 \times PNM_4$	29.90	29.05	32.95	35.25	37.25	36.50
$I_3 \times PNM_5$	24.25	30.69	29.36	37.00	38.60	32.56
Control	28.25	25.65	29.25	36.25	38.20	38.25

For detailed description of I (Irrigation) and PNM (Precision nutrient management) refer main table 2

Table 5 **NDVI at weekly intervals between 30-45 DAS and 60-90 DAS during 2020**

Treatments	30-45 DAS			60-90 DAS		
	1st week	2nd week	3rd week	1st week	2nd week	3rd week
$I_1×PNM_1$	0.37	0.36	0.37	0.55	0.54	0.54
$I_1×PNM_2$	0.36	0.35	0.35	0.53	0.57	0.55
$I_1×PNM_3$	0.45	0.46	0.39	0.55	0.55	0.56
$I_1×PNM_4$	0.36	0.37	0.33	0.54	0.54	0.55
$I_1×PNM_5$	0.37	0.37	0.36	0.48	0.49	0.46
$I_2×PNM_1$	0.52	0.49	0.51	0.67	0.71	0.65
$I_2×PNM_2$	0.49	0.48	0.47	0.59	0.69	0.65
$I_2×PNM_3$	0.54	0.55	0.56	0.72	0.73	0.73
$I_2×PNM_4$	0.42	0.46	0.47	0.66	0.66	0.63
$I_2×PNM_5$	0.38	0.42	0.41	0.56	0.62	0.63
$I_3×PNM_1$	0.40	0.41	0.43	0.61	0.60	0.60
$I_3×PNM_2$	0.40	0.41	0.42	0.62	0.59	0.57
$I_3×PNM_3$	0.42	0.43	0.45	0.62	0.59	0.58
$I_3×PNM_4$	0.41	0.43	0.39	0.61	0.58	0.56
$I_3×PNM_5$	0.38	0.36	0.41	0.58	0.57	0.57
Control	0.42	0.39	0.31	0.52	0.58	0.47

For detailed description of I (Irrigation) and PNM (Precision nutrient management) refer main table 2

Table 6 **NDVI at weekly intervals between 30-45 DAS and 60-90 DAS during 2021**

Treatments	30-45 DAS			60-90 DAS		
	1st week	2nd week	3rd week	1st week	2nd week	3rd week
$I_1×PNM_1$	0.37	0.35	0.45	0.53	0.65	0.65
$I_1×PNM_2$	0.36	0.41	0.38	0.55	0.65	0.65
$I_1×PNM_3$	0.45	0.44	0.47	0.58	0.60	0.60
$I_1×PNM_4$	0.33	0.32	0.34	0.48	0.66	0.66
$I_1×PNM_5$	0.35	0.35	0.24	0.45	0.48	0.48
$I_2×PNM_1$	0.49	0.53	0.48	0.71	0.68	0.68
$I_2×PNM_2$	0.44	0.39	0.48	0.64	0.62	0.68
$I_2×PNM_3$	0.53	0.50	0.58	0.71	0.72	0.72
$I_2×PNM_4$	0.43	0.42	0.49	0.66	0.70	0.70
$I_2×PNM_5$	0.38	0.37	0.45	0.62	0.62	0.62
$I_3×PNM_1$	0.46	0.48	0.41	0.60	0.69	0.69
$I_3×PNM_2$	0.47	0.39	0.39	0.56	0.64	0.70
$I_3×PNM_3$	0.43	0.35	0.36	0.57	0.66	0.66
$I_3×PNM_4$	0.46	0.41	0.43	0.54	0.65	0.65
$I_3×PNM_5$	0.43	0.35	0.36	0.57	0.66	0.58
Control	0.37	0.46	0.42	0.48	0.49	0.52

For detailed description of I (Irrigation) and PNM (Precision nutrient management) refer main table

Table 7 Canopy temperature depression (CTD) at weekly intervals between 30-45 DAS and 60-90 DAS during 2020

Treatments	30-45 DAS			60-90 DAS		
	1st week	2nd week	3rd week	1st week	2nd week	3rd week
$I_1 \times PNM_1$	3.11	2.87	4.12	4.78	3.17	2.75
$I_1 \times PNM_2$	3.12	3.50	3.22	3.24	3.05	2.11
$I_1 \times PNM_3$	3.90	3.07	3.45	3.87	3.11	3.20
$I_1 \times PNM_4$	2.90	2.50	3.72	3.53	3.52	2.23
$I_1 \times PNM_5$	3.01	3.00	3.01	3.23	2.86	2.11
$I_2 \times PNM_1$	3.92	4.30	4.20	4.23	4.92	4.33
$I_2 \times PNM_2$	4.01	4.10	4.06	4.22	4.19	4.32
$I_2 \times PNM_3$	4.09	4.05	4.25	4.65	4.22	4.83
$I_2 \times PNM_4$	3.98	3.75	4.26	4.81	4.21	4.38
$I_2 \times PNM_5$	3.01	4.50	3.85	4.49	4.23	4.53
$I_3 \times PNM_1$	3.98	3.75	4.26	5.62	3.21	3.22
$I_3 \times PNM_2$	3.82	3.92	3.72	3.40	3.45	3.55
$I_3 \times PNM_3$	3.99	4.16	4.26	4.53	4.37	4.46
$I_3 \times PNM_4$	3.56	3.56	3.96	3.10	4.04	3.70
$I_3 \times PNM_5$	3.29	3.96	3.25	3.01	4.14	3.25
Control	3.31	3.20	3.30	3.50	3.20	3.65

For detailed description of I (Irrigation) and PNM (Precision nutrient management) refer main table2

Table 8 Canopy temperature depression (CTD) at weekly intervals between 30-45 DAS and 60-90 DAS during 2021

Treatments	30-45 DAS			60-90 DAS		
	1st week	2nd week	3rd week	1st week	2nd week	3rd week
$I_1 \times PNM_1$	3.98	3.81	3.90	2.97	3.15	3.23
$I_1 \times PNM_2$	3.78	3.67	3.70	3.04	3.06	3.09
$I_1 \times PNM_3$	3.98	3.78	3.68	3.11	3.06	3.39
$I_1 \times PNM_4$	3.67	3.45	3.75	4.07	2.05	3.08
$I_1 \times PNM_5$	3.55	3.25	3.55	2.86	3.18	3.01
$I_2 \times PNM_1$	4.50	4.30	4.98	4.36	4.14	4.22
$I_2 \times PNM_2$	4.61	4.52	4.78	3.94	4.19	3.95
$I_2 \times PNM_3$	3.98	5.45	4.55	4.29	4.37	4.24
$I_2 \times PNM_4$	4.52	4.48	4.56	4.26	3.93	4.26
$I_2 \times PNM_5$	4.69	4.75	5.26	3.97	3.96	4.08
$I_3 \times PNM_1$	3.80	3.65	3.94	3.75	3.73	3.56
$I_3 \times PNM_2$	3.75	3.66	3.89	3.46	3.44	3.22
$I_3 \times PNM_3$	4.20	3.50	4.20	4.02	3.72	4.26
$I_3 \times PNM_4$	3.98	3.76	3.89	3.44	3.37	3.35
$I_3 \times PNM_5$	3.78	3.86	3.98	3.21	3.19	3.23
Control	3.61	3.20	3.56	2.60	3.00	2.69

For detailed description of I (Irrigation) and PNM (Precision nutrient management) refer main table

Table 9. Effect of precision nutrient and irrigation management on grain and stover yield of soybean

**Treatment**	**t ha^{-1}**			
	**Grain yield**		**Biological yield**	
	**2020**	**2021**	**2020**	**2021**
Irrigation				
I_1	1.99	1.97	6.69	6.51
I_2	2.45	2.56	7.61	7.72
I_3	2.15	2.30	6.34	6.65
SEm ±	0.03	0.10	0.10	0.17
CD (P=0.05)	0.14	0.39	0.40	0.65
Precision nutrient management				
PNM_1	2.27	2.34	7.06	7.13
PNM_2	2.17	2.29	6.71	6.70
PNM_3	2.45	2.43	7.67	7.65
PNM_4	2.20	2.12	6.91	6.96
PNM_5	1.89	2.20	6.05	6.36
SE m ±	0.03	0.06	0.08	0.08
CD (P=0.05)	0.08	0.19	0.24	0.25
SCI versus control				
SCI	2.20	2.28	6.88	6.96
C	1.95	1.80	6.75	6.88
SE m ±	0.05	0.03	0.10	0.02
CD(P=0.05)	0.13	0.08	NS	0.06

For detailed description of I (Irrigation) and PNM (Precision nutrient management) refer main table

Table 10 Interaction effect of precision nutrient and irrigation management on irrigation water productivity, economic water productivity and water use efficiency of soybean

Treatment	IWP Kg ha^{-1} m^{-3}		EWP INR ha^{-1} m $^{-3}$		WUE Kg ha–mm^{-1}day^{-1}	
	2020	2021	2020	2021	2020	2021
I_1×PNM_1	0.55$^{(*)}$	0.69$^{(*)}$	18.1$^{(*)}$	13.7$^{(*)}$	6.33$^{(*)}$	6.59$^{(*)}$
I_1×PNM_2	0.51$^{(*)}$	0.62$^{(*)}$	16.5$^{(*)}$	12.4$^{(*)}$	5.79$^{(*)}$	5.89$^{(*)}$
I_1×PNM_3	0.62$^{(*)}$	0.66$^{(*)}$	20.2$^{(*)}$	13.4$^{(*)}$	7.09$^{(*)}$	6.32$^{(*)}$
I_1×PNM_4	0.58$^{(*)}$	0.64$^{(*)}$	18.3$^{(*)}$	12.6$^{(*)}$	6.58$^{(*)}$	6.06$^{(*)}$
I_1×PNM_5	0.45$^{(NS)}$	0.67$^{(*)}$	15.1$^{(NS)}$	13.1$^{(*)}$	5.18$^{(NS)}$	6.37$^{(*)}$
I_2×PNM_1	0.92$^{(*)}$	0.99$^{(*)}$	25.7$^{(*)}$	17.8$^{(*)}$	7.34$^{(*)}$	7.95$^{(*)}$
I_2×PNM_2	0.90$^{(*)}$	1.03$^{(*)}$	25.2$^{(*)}$	18.3$^{(*)}$	7.20$^{(*)}$	8.26$^{(*)}$
I_2×PNM_3	0.93$^{(*)}$	1.06$^{(*)}$	26.4$^{(*)}$	18.9$^{(*)}$	7.46$^{(*)}$	8.45$^{(*)}$
I_2×PNM_4	0.88$^{(*)}$	1.03$^{(*)}$	25.0$^{(*)}$	18.3$^{(*)}$	7.05$^{(*)}$	8.26$^{(*)}$
I_2×PNM_5	1.04$^{(*)}$	0.96$^{(*)}$	21.6$^{(*)}$	17.2$^{(*)}$	6.24$^{(*)}$	7.67$^{(*)}$
I_3×PNM_1	0.99$^{(*)}$	1.28$^{(*)}$	24.1$^{(*)}$	18.7$^{(*)}$	5.91$^{(*)}$	7.70$^{(*)}$
I_3×PNM_2	0.96$^{(*)}$	1.27$^{(*)}$	23.7$^{(*)}$	18.6$^{(*)}$	5.78$^{(*)}$	7.64$^{(*)}$
I_3×PNM_3	1.11$^{(*)}$	1.39v	27.0$^{(*)}$	20.2$^{(*)}$	6.65$^{(*)}$	8.37$^{(*)}$
I_3×PNM_4	0.90$^{(*)}$	0.97$^{(*)}$	22.1$^{(*)}$	14.7$^{(*)}$	5.38$^{(*)}$	5.81$^{(NS)}$
I_3×PNM_5	0.81$^{(*)}$	1.15$^{(*)}$	20.3$^{(*)}$	17.0$^{(*)}$	4.89$^{(*)}$	6.90
PNM means at same level of I						
SEm ±	0.021	0.033	0.46	0.61	0.11	0.13
CD (0.05)	0.062	0.098	1.36	1.77	0.31	0.39
I means at same or different level of PNM						
SEm ±	0.026	0.050	0.56	0.78	0.12	0.16
CD (0.05)	0.086	0.177	1.88	2.68	0.41	0.52
Pair-wise Comparison of control vs rest of the individual combinations						
C	0.46	0.43	15.8	10.8	5.62	5.7
SEm ±	0.005	0.009	0.12	0.16	0.03	0.09
CD (P=0.05)	0.016	0.025	0.35	0.46	0.10	0.27

For detailed description of I (Irrigation) and PNM (Precision nutrient management) refer main table. (*) parenthesis indicates I×PNM combination under SCI are significant ($p<0.05$) over Control whereas (ns) indicates non-significant difference between C and I×PNM.
